# Monomodular *Pseudomonas aeruginosa* phage JG004 lysozyme (Pae87) contains a bacterial surface-active antimicrobial peptide-like region and a possible substrate-binding subdomain

**DOI:** 10.1107/S2059798322000936

**Published:** 2022-03-04

**Authors:** Roberto Vázquez, Mateo Seoane-Blanco, Virginia Rivero-Buceta, Susana Ruiz, Mark J. van Raaij, Pedro García

**Affiliations:** a Microbial and Plant Biotechnology Department, Centro de Investigaciones Biológicas Margarita Salas (CIB-CSIC), Madrid, Spain; b Centro de Investigación Biomédica en Red de Enfermedades Respiratorias (CIBERES), Madrid, Spain; c Department of Macromolecular Structure, Centro Nacional de Biotecnología (CNB-CSIC), Madrid, Spain; d Interdisciplinary Platform for Sustainable Plastics, Madrid, Spain

**Keywords:** endolysins, phage lysins, Pae87, bacteriophages, antimicrobial peptides, protein structure, *Pseudomonas aeruginosa*, carbohydrate-active enzymes

## Abstract

Phage lysins are a source of novel antimicrobials to tackle the bacterial antibiotic-resistance crisis. The engineering of phage lysins is being explored as a game-changing technological strategy to introduce a more precise approach in the way in which antimicrobial therapy is applied. Such engineering efforts will benefit from a better understanding of lysin structure and function. In this work, the antimicrobial activity of the endolysin from *Pseudomonas aeruginosa* phage JG004, termed Pae87, has been characterized. This lysin had previously been identified as an antimicrobial agent candidate that is able to interact with the Gram-negative surface and disrupt it. Further evidence is provided here based on a structural and biochemical study. A high-resolution crystal structure of Pae87 complexed with a peptidoglycan fragment showed a separate substrate-binding region within the catalytic domain, 18 Å away from the catalytic site and located on the opposite side of the lysin molecule. This substrate-binding region was conserved among phylogenetically related lysins lacking an additional cell-wall-binding domain, but not among those containing such a module. Two glutamic acids were identified to be relevant for the peptidoglycan-degradation activity, although the antimicrobial activity of Pae87 was seemingly unrelated. In contrast, an antimicrobial peptide-like region within the Pae87 C-terminus, named P87, was found to be able to actively disturb the outer membrane and display antibacterial activity by itself. Therefore, an antimicrobial mechanism for Pae87 is proposed in which the P87 peptide plays the role of binding to the outer membrane and disrupting the cell-wall function, either with or without the participation of the catalytic activity of Pae87.

## Abstract 2

The structure of the monomodular *Pseudomonas aeruginosa* bacteriophage JG004 lysin Pae87 is presented and investigated in relation to repurposing its function as an antimicrobial agent. The structure with its peptidoglycan ligand revealed a possible cell-wall-binding region. A C-terminal antimicrobial peptide-like region is shown to be important for disrupting the bacterial cell wall.

## 1. Introduction

Antibiotic resistance is becoming one of the most serious threats to public health worldwide, as the number of multi-resistant bacterial strains is growing progressively. Therefore, the search for alternative treatments to standard antibiotics to fight these pathogenic ‘superbugs’ is becoming more urgent (O’Neill, 2016 ▸). Bacteriophage (phage) lysins are a promising strategy that is currently viewed as a feasible approach towards the development of novel, potentially marketable antibacterial agents (Abdelkader *et al.*, 2019 ▸). Lysins are highly evolved enzymes produced by phages at the end of the lytic infection cycle to degrade the bacterial peptidoglycan, leading to cell lysis and phage progeny release. For about twenty years, phage lysins have been widely investigated as novel antimicrobial agents to treat bacterial infections mainly caused by Gram-positive pathogens. This mechanism relies on adding the purified enzyme exogenously (‘lysis from without’) and provokes the rapid degradation of the substrate (the peptidoglycan) and thus the lysis and death of susceptible bacteria, including multi-drug-resistant strains. Lysins have demonstrated several advantages over standard antibiotics, including (i) rapid killing activity against both stationary- and exponential-phase bacteria, practically within a few minutes of contact with the peptidoglycan substrate, (ii) effectiveness against multi-drug-resistant bacteria, (iii) specificity to the target pathogen, especially against Gram-positive bacteria, which allows the preservation of the normal microbiota, (iv) a seemingly very unlikely appearance of resistance, probably due to the conservation of its substrate, the peptidoglycan, (v) synergistic effects with other lysins or antibiotics and (vi) efficient lethal activity against colonizing pathogens growing on mucosal surfaces and/or in biofilms (Pastagia *et al.*, 2013 ▸).

The architecture of lysins varies depending on their origin, but those from phages infecting Gram-positive bacteria usually have a modular structure consisting of one or two catalytic domains, which harbour lytic activity against the host species, and a C-terminal cell-wall-binding domain (CWBD), which recognizes a cell-wall traits specific to the bacteria that it targets. On the contrary, the great majority of lysins from phages infecting Gram-negative bacteria display a globular organization containing only one catalytic domain (Vázquez, García *et al.*, 2021 ▸). The catalytic domains are responsible for the cleavage of a specific bond within the peptidoglycan, and based on the bond that they break lysins can be classified as glycosidases, *N*-acetylglucosamine amidases (NAM-amidases) or endopeptidases (Dams & Briers, 2019 ▸). Among glycosidase lysins, which are those that act within the glycan strand of *N*-acetylglucosamine (NAG) and *N*-acetylmuramic acid (MurNAc), two subclasses can be recognized. *N*-Acetyl­muramidases, which are termed muramidases or lysozymes, hydrolyze the bond between MurNAc and NAG at the reducing side of the former, while *N*-acetylglucosaminidases break the bond between NAG and MurNAc at the reducing side of NAG. The CWBDs that many lysins bear are responsible for the specific recognition of the insoluble substrate and the high-affinity binding of these enzymes to susceptible bacteria (Guillén *et al.*, 2010 ▸; Low *et al.*, 2011 ▸).

In a previous study (Vázquez, Blanco-Gañán *et al.*, 2021 ▸), the lysin from *Pseudomonas aeruginosa* phage JG004, termed Pae87, was mined from a data set of phage lysin sequences (Vázquez, García *et al.*, 2021 ▸; Vázquez *et al.*, 2020 ▸) on the basis of containing a putative C-terminal positively charged, antimicrobial peptide (AMP)-like region. The presence of such a region was probed under the hypothesis that it would enable Pae87 to disrupt the Gram-negative outer membrane (OM), which is often cited as a barrier to lysin activity from without (Briers & Lavigne, 2015 ▸). Pae87 was demonstrated to have an antimicrobial effect against *P. aeruginosa* and some other Gram-negative pathogens (Vázquez, Blanco-Gañán *et al.*, 2021 ▸). Nowadays, insightful knowledge into the structure of lysins, as well as implications for their function (either as exogenous antimicrobials or as lysis effectors for the release of virion particles from the host cell), is a must in order to engage in protein-based antimicrobial engineering. An example is the combinatorial engineering of lysin modules or the derivation of AMPs from lysins (Duyvejonck *et al.*, 2021 ▸; Thandar *et al.*, 2016 ▸). Therefore, in this work we provide a fine characterization of the structural elements that contribute to the observed activities of Pae87. We do so by (i) obtaining the crystal structure of the protein, (ii) examining putative catalytic residues by point mutation and (iii) testing the antimicrobial and membrane-permeabilizing activity of Pae87 and the AMP derived from its C-terminal region, named P87.

## 2. Materials and methods

### 2.1. Bacterial strains, media and growth conditions

All of the Gram-negative bacteria used in this work (*P. aeruginosa*, *Escherichia coli*, *Acinetobacter baumannii*, *Acinetobacter pitti* and *Klebsiella pneumoniae*) were grown in lysogeny broth (LB, NZYTech) at 37°C with aeration (shaking at 200 rev min^−1^), except for *Moraxella catarrhalis*. This bacterial species, plus some Gram-positive species (*Staphylococcus aureus*, *Streptococcus pyogenes* and *Streptococcus* Milleri group strain), were cultured in brain heart infusion broth (BHI, Condalab) at 37°C. *M. catarrhalis* and *S. aureus* were shaken at 200 rev min^−1^ when grown in liquid culture. *Streptococcus pneumoniae* was grown in C medium adjusted to pH 8.0 (Lacks & Hotchkiss, 1960 ▸) supplemented with 0.08% yeast extract (C+Y) at 37°C without shaking. For solid cultures, all Gram-positive species and *M. catarrhalis* were grown on blood agar plates, while Gram-negative species were grown on LB agar. Details of the bacterial strains used in this work can be found in Vázquez, Blanco-Gañán *et al.* (2021 ▸).

### 2.2. Plasmids, oligonucleotides and overlap extension mutagenesis

The plasmids and oligonucleotides used throughout this work are given in Supplementary Table S1. As explained in Vázquez, Blanco-Gañán *et al.* (2021 ▸), a synthetic gene encoding Pae87 (*pae87*) was obtained from GenScript and was cloned into a pET-28a(+) vector in frame with an N-terminal 6×His tag. This expression plasmid, named pET-PA87, was heat-shock-transformed into *E. coli* BL21(DE3) cells. For the construction of *pae87* mutants (E29A, E46A and E29A/E46A), an overlap extension PCR protocol was performed. Briefly, the pET-PA87 plasmid was used as a template for two separate PCR reactions per mutation. Each of these reactions amplified a fragment of the *pae87* gene in such a way that both fragments shared an overlapping section (∼20 nt) in which the mutated bases were located. A third PCR reaction was then performed using the *pae87* flanking primers pae87_f and pae87_3′ and a mixture of the resulting amplification products from the previous step as a template. The final PCR products were purified, digested with NdeI and HindIII, cloned into a pre-digested pET-28a(+) vector and transformed into *E. coli* DH10B cells. Colonies were screened by PCR and vectors putatively bearing the mutated gene were sequenced (Secugen, Centro de Investigaciones Biológicas Margarita Salas, Madrid, Spain). The final vectors were transformed into *E. coli* BL21(DE3) cells for expression.

### 2.3. Protein expression and purification

For the production of recombinant Pae87-based proteins, the appropriate strains were cultured in 1 l LB in the presence of 50 µg ml^−1^ kanamycin to an OD_600_ of ∼0.6–0.8. Expression was then induced with 0.4 m*M* isopropyl β-d-1-thiogalactopyranoside and incubation was resumed at 20°C for up to 48 h. After centrifugation (12 000*g*, 20 min, 4°C), the pelleted biomass was resuspended in ∼30 ml 20 m*M* sodium phosphate buffer (NaPiB) pH 7.4 containing 0.3 *M* NaCl and 40 m*M* imidazole. These cell suspensions were disrupted by sonication and the cell debris was separated again by centrifugation (18 000*g*, 20 min, 4°C). The supernatants containing the protein extracts were then applied onto a HisTrap FF 5 ml column (GE Healthcare) loaded with nickel ions using an ÄKTA start liquid-chromatography machine (GE Healthcare). After a thorough washing step using the resuspension buffer, 20 m*M* NaPiB pH 7.4 containing 0.3 *M* NaCl and 0.5 *M* imidazole was used to elute the purified protein. The buffer of the purified fractions was exchanged to 20 m*M* NaPiB pH 7.4 containing 150 m*M* NaCl prior to assaying the proteins using HiTrap Desalting 5 ml columns. The concentration of the purified proteins was estimated from *A*_280_ measurements using predicted molar extinction coefficients (Supplementary Table S2). Protein samples were maintained at 4°C for up to a month without apparent signs of precipitation or loss of activity.

### 2.4. Synthesis and quantification of peptide P87

Peptide P87 (LNTFVRFIKINPAIHKALKSKNWAEFAKR) was synthesized and provided by GenScript as a freeze-dried powder. It was dissolved in distilled water and the concentration was estimated by measuring the *A*_280_ using a molar extinction coefficient of 5500 *M* ^−1^ cm^−1^ as predicted by *ProtParam* (Wilkins *et al.*, 1999 ▸). Diluted peptide aliquots were kept at −20°C.

### 2.5. *P. aeruginosa* PAO1 peptidoglycan purification and muralytic activity assay

*P. aeruginosa* PAO1 peptidoglycan purification and a dye-release muralytic activity assay were conducted essentially as described in Vázquez, Blanco-Gañán *et al.* (2021 ▸). Briefly, *P. aeruginosa* PAO1 cells were cultured in 1 l LB broth until the OD_600_ reached 0.8–1.0. The culture was then centrifuged (4000*g*, 15 min, 4°C) and resuspended in 20 ml PBS. 80 ml 5% SDS was added and the mixture was boiled for 30 min with vigorous shaking. After overnight incubation at room temperature, the suspension was ultracentrifuged (100 000*g* for 60 min at 20°C) and the pellet was resuspended in distilled water and subjected to dialysis against water for 24–72 h to wash out as much SDS as possible. The samples were then ultracentrifuged in the same conditions as before and washed again as many times as necessary to remove all SDS (typically 1–3 more times, until a thick foam layer was no longer formed upon resuspension). RNA, DNA and proteins were then eliminated by successive treatments with RNAse, DNAse and trypsin as described in Vázquez, Blanco-Gañán *et al.* (2021 ▸). Finally, the peptidoglycan sacculi were ultracentrifuged again, the supernatant was removed and the pellet was dried for 24–48 h at 37°C to determine the dry weight yield of the process (typically 12 mg per litre of initial culture).

Purified sacculi were dyed by resuspending them in freshly prepared 0.02 *M* Remazol Brilliant Blue (RBB) solution in 0.2 *M* sodium hydroxide. An incubation of about 6 h was conducted at 37°C with shaking followed by overnight incubation at 4°C. After staining, several ultracentrifugation and washing steps with distilled water were conducted until the supernatants were clear (usually 3–4 washing steps). The resuspension water volume of the final pellets was adjusted to an *A*_595_ of ∼1.5. For the dye-release assay, 100 µl of the RBB-stained sacculi were centrifuged (12 000*g*, 20 min, 20°C) and the supernatant was discarded. The pelleted sacculi were then resuspended in 100 µl of a solution of NaPiB pH 6.0 containing 150 m*M* NaCl and the desired concentration of enzyme or just buffer for the control. The samples were incubated for 10 min at 37°C and reactions were stopped by incubation for a further 5 min at 95°C. The samples were then centrifuged (12 000*g*, 20 min, 20°C) and the *A*_595_ of the supernatants was determined using a VersaMax multi-well plate spectrophotometer (Molecular Devices).

### 2.6. Antimicrobial activity assays

Antimicrobial activity assays were performed by incubating a resting bacterial cell suspension in NaPiB pH 6.0 with 150 m*M* NaCl at 37°C together with the corresponding antibacterial protein or peptide (Vázquez, Blanco-Gañán *et al.*, 2021 ▸). Bacteria at the mid-to-late exponential phase, as evaluated by turbidimetry, were harvested by centrifugation (3000*g*, 10 min, 4°C). The pelleted cells were resuspended in half the volume of buffer and pipetted onto a 96-well plate (100 µl per well). 100 µl of the same buffer containing the desired concentration of the compound to be tested was then added and the plate was incubated at 37°C for 2 h. Several measurements were performed on the treated resting cells, namely OD_600_ monitoring, viable cell counts by plating tenfold serial dilutions at the end of the experiment and observation under a fluorescence microscope [Leica DM4000B with an HC PL APO 100×/1.40 oil objective and L5 (bandpass 480/40) and N2.1 (515/60) filters] stained with the BacLight LIVE/DEAD bacterial viability kit L-13152 (Invitrogen–Molecular Probes, containing SYTO9 and propidium iodide).

### 2.7. Analysis of degradation products

To analyze the degradation products that resulted from Pae87 activity, a similar protocol to that described in Alvarez *et al.* (2016 ▸) was followed. 100 µl of the *P. aeruginosa* PAO1 purified peptidoglycan was centrifuged (12 000*g*, 20 min, room temperature) and resuspended again in the same volume of a suitable reaction buffer (20 m*M* NaPiB pH 6.5, 100 m*M* NaCl for Pae87 or 50 m*M* NaPiB pH 4.9 for the positive control cellosyl). 10 µg of the corresponding enzyme (or an equivalent volume of water for the negative control) was then added and the samples were incubated overnight at 37°C. Reactions were stopped by incubation at 98°C for 5 min. The tubes were then centrifuged (12 000*g*, 20 min, room temperature) and the supernatants were collected. 0.5 *M* borate buffer pH 9.0 was added to adjust the pH of the samples to 8.5–9.0 and 10 µl of freshly prepared 2 *M* NaBH_4_ were added to reduce the sample at room temperature for 30 min. Next, the pH was adjusted to 2.0–4.0 with 25% orthophosphoric acid. The soluble muropeptides of each sample were then separated by reverse-phase high-performance liquid chromatography (RP-HPLC) on a Kinetex C18 Column (1.7 µm, 100 Å, 150 × 2.1 mm, Phenomenex) coupled to an LXQ mass spectrometer equipped with a linear ion trap (Finnigan LXQ, Thermo Scientific). Ionization was achieved by electrospray. Muropeptides were eluted at a flow rate of 0.4 ml min^−1^ with the following elution gradient: *t* = 0 min, 95% *A*; *t* = 0.5 min, 93% *A*; *t* = 3 min, 82% *A*; *t* = 11 min, 50% *A*; *t* = 12 min, 50% *A*; *t* = 12.1 min, 95% *A*; *t* = 15 min, 95% *A*; *A* is 0.1% formic acid in water and *B* is 0.1% formic acid in 40% acetonitrile. Mass spectrometry (MS) was performed using a double-play mode in which the instrument was set to acquire a full MS scan (*m*/*z* = 150–2000) and a collision-induced dissociation (CID) spectrum on selected ions from the full MS scan. Spectra were analyzed using the *Xcalibur* software (Thermo Scientific).

### 2.8. Pae87 production and crystallization

*E. coli* BL21(DE3) cells transformed with pET-PA87 were grown at 37°C as described in Section 2.3. After reaching an OD_600_ of about 0.6, cultures were cooled and Pae87 expression was induced with 0.5 m*M* isopropyl β-d-1-thiogalactopyranoside at 20°C overnight. The cells were centrifuged at 6000*g* for 10 min at 4°C and the pellets were stored frozen. Later, the pellets from 1 l culture were resuspended in 10 ml lysis buffer (20 m*M* Tris pH 7.5, 0.5 *M* NaCl, 20 m*M* imidazole, 5% glycerol). The cells were then disrupted with a Digital Sonifier 250 (Branson) and centrifuged at 15 000*g* for 45 min at 4°C. Supernatants from centrifugation of the disrupted cells were incubated with 2 ml nickel–nitrilotriacetic acid (Ni–NTA) agarose (Jena Bioscience) on ice for 30 min. The mixture was then poured into an Econo-Pac Chromatography Column (Bio-Rad) and eluted by gravity. After a two-column-volume wash with lysis buffer, the protein was eluted by passing 2 ml elution buffer (20 m*M* Tris pH 7.5, 0.5 *M* NaCl, 0.5 m*M* imidazole, 5% glycerol) six times. After analysis by denaturing gel electrophoresis, the protein-containing fractions were dialyzed in a cellulose membrane tube (14 kDa molecular-weight cutoff, Merck–Millipore), first against 1 l 20 m*M* Tris–HCl pH 7.5, 0.2 *M* NaCl for 3–4 h at 4°C and then against 1 l 20 m*M* Tris–HCl pH 7.5 overnight at 4°C. Prior to ion-exchange chromatography, the dialyzed sample was centrifuged at 15 000*g* for 10 min at 4°C to remove aggregates. Chromatography was performed using an ÄKTApurifier 10 FPLC system (Cytiva) with a RESOURCE Q 6 ml column. Proteins were eluted in a gradient of 0–1 *M* NaCl buffer with 20 m*M* Tris–HCl pH 7.5. Pae87 eluted at approximately 0.18 *M* NaCl. Fractions containing pure Pae87 were desalted and concentrated using Amicon Ultra centrifugal filters with 3 or 10 kDa molecular-weight cutoff (Millipore). Concentrated samples were centrifuged at 15 000*g* for 10 min prior to crystallization trials; the pellet was discarded. The Pae87 concentration was estimated by measuring the *A*_280_ using the predicted molar extinction coefficient (Supplementary Table S2).

Proteins were crystallized using the sitting-drop vapour-diffusion technique in MCR crystallization plates (SWISSCI). Reservoirs were filled with 50 µl of various crystallization solutions. Drops consisted of 1 µl protein solution (in 20 m*M* Tris–HCl pH 7.5) plus 0.5 µl crystallization solution. The best apoprotein crystal was obtained at 11 mg ml^−1^ Pae87 when 20%(*w*/*v*) polyethylene glycol 8000 and 0.1 *M* CHES–NaOH pH 9.5 was used as the crystallization solution. For the ligand–protein complex crystallization assay, 3 mg of the dried *P. aeruginosa* PAO1 peptidoglycan were solubilized by adding 120 µl of the purified Pae7 protein at 11 mg ml^−1^ in 20 m*M* Tris–HCl pH 7.5. The mixture was centrifuged at 15 000*g* for 10 min at 4°C to remove aggregates. The supernatant was then used in crystallization trials. The best crystal appeared when 0.1 m*M* Tris–HCl pH 8.0, 20%(*w*/*v*) polyethylene glycol monomethyl ether 5000, 8%(*v*/*v*) ethylene glycol was used as the crystallization solution.

### 2.9. Crystallographic data collection, structure determination and analysis

Diffraction data were obtained on the XALOC beamline at the ALBA-CELLS synchrotron, Cerdanyola del Vallès, Spain (Juanhuix *et al.*, 2014 ▸) using a Dectris PILATUS 6M detector. Diffraction images were processed using the *CCP*4 suite (Winn *et al.*, 2011 ▸). *xia*2/*DIALS* (Winter, 2010 ▸) was used to index and integrate the images, *POINTLESS* (Evans, 2006 ▸) was used for space-group determination and *AIMLESS* (Evans & Murshudov, 2013 ▸) was used for scaling. The crystal structure of the apo form was determined by molecular replacement with *MOLREP* (Vagin & Teplyakov, 2010 ▸) using PDB entry 5nm7 as the search model (Maciejewska *et al.*, 2017 ▸). The protein model was modified using the graphics program *Coot* (Emsley *et al.*, 2010 ▸) and was refined with *REFMAC*5 (Murshudov *et al.*, 2011 ▸). The protein–ligand complex structure was similarly obtained by molecular replacement using molecule *A* of the apo-form model as the search model. The resolution limit for the data to be included was determined by paired refinement with *PDB_REDO* (Joosten *et al.*, 2014 ▸). Models were validated with *MolProbity* (http://molprobity.biochem.duke.edu/; Williams *et al.*, 2018 ▸). *PyMOL* (version 1.8; Schrödinger) was used for analysis and visualization of the models. Structure comparison was performed using the *DALI* server (Holm, 2020 ▸) and oligomerization parameters (accessible and buried surfaces, estimated dissociation energies) were analyzed with *QtPISA* (Krissinel, 2015 ▸).

### 2.10. Bioinformatic analyses

The lysin sequence data set published in Vázquez, García *et al.* (2021 ▸) and available from Vázquez *et al.* (2020 ▸) was used as a sample for various analyses. Multiple sequence alignments were performed using *Clustal Omega* as implemented at the EMBL–EBI (Madeira *et al.*, 2019 ▸) and were visualized using *JalView* (Waterhouse *et al.*, 2009 ▸). *HeliQuest* (Gautier *et al.*, 2008 ▸) and *EMBOSS: charge* (Rice *et al.*, 2000 ▸) were used to predict the physicochemical properties of protein helices and the local charge plots of amino-acid sequences. To specifically determine the sequence of the AMP-like fragment at the C-terminus of Pae87, a score variable was calculated consisting of the half-sum of the min–max standardized net charge (NC) and hydrophobic moment (HM) for each 11-amino acid fragment of Pae87,

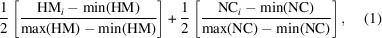

where HM_*i*_ and NC_*i*_ are specific values of HM or NC, respectively, and HM and NC are the respective sets of values for each variable. In this way, the score measured the magnitude of both parameters with equal weight and on a 0–1 scale.

Sequence-similarity networks (SSNs) were generated to visually assess the similarity clustering of sequence sets. For this purpose, the *Enzyme Similarity Tool* from the *Enzyme Function Initiative* server (*EFI-EST*) was employed (Zallot *et al.*, 2019 ▸). Briefly, this tool performs a local alignment, from which every possible pair of sequences receives a score similar to the *E*-value obtained from a typical *BLAST* analysis. A threshold score value was selected for the SSN so that below such a threshold sequence pairs were considered to be non­similar and therefore the pair would not be connected in the resulting representation. The score was selected so that sequence pairs with a similarity below 30–40% were deemed to be nonsimilar. The SSN graphs were produced using *Cytoscape* 3 with identity percentage-based Prefuse Force Directed layout (Shannon *et al.*, 2003 ▸).

### 2.11. Circular dichroism

Circular-dichroism spectra were acquired at 4°C in 20 m*M* NaPiB pH 6.5, 150 m*M* NaCl using a Jasco J700 spectropolarimeter equipped with a temperature-controlled holder. Far-UV spectra were recorded from 260 to 200 nm in a 1 mm path-length quartz cuvette. Each spectrum was obtained by averaging five accumulations collected at a scan rate of 50 nm min^−1^ with 2 s response time. Buffer spectra were subtracted from 0.1 mg ml^−1^ peptide spectra and the molar ellipticity per residue was calculated. Far-UV spectra collected with different concentrations of 2,2,2-trifluoroethanol (TFE) were used to predict the ability of peptides to form secondary structures in the presence of biological membranes.

### 2.12. Outer membrane (OM) permeabilization assay

The physiological effects of the interaction of proteins or peptides with the *P. aeruginosa* PAO1 OM were assayed using *N*-phenyl-1-naphthylamine (NPN) as a fluorescent probe (Loh *et al.*, 1984 ▸; Helander & Mattila-Sandholm, 2000 ▸). The assay was conducted using suspensions of PAO1 resting cells prepared from an actively growing culture at ∼10^8^ CFU per millitre pelleted (3000*g*, 10 min, 20°C) and resuspended in half the volume of 20 m*M* NaPiB pH 6.0, 150 m*M* NaCl, 100 m*M* sorbitol. The sorbitol was added as an additional osmoprotectant to maintain cellular integrity. 100 µl of this suspension was added to each well of a FluoroNunc 96-well plate together with 50 µl of 40 µ*M* NPN and 50 µl of the enzyme or peptide at the corresponding concentration, or just buffer for the negative control. Fluorescence was then immediately recorded in a Varioskan Flash microplate reader (Thermo Scientific) with an excitation wavelength of 350 nm and a fluorescence-detection wavelength of 420 nm. Nondyed PAO1 cells treated with just buffer were also incorporated as a background measurement and EDTA was used as a positive control for OM permeabil­ization. Samples were also observed by fluorescence microscopy using an A filter cube (Leica) with a bandpass of 340–380 nm.

### 2.13. Fluorochrome labelling of Pae87

An *N*-hydroxysuccinimidyl (NHS) ester of Alexa488 fluorochrome (NHS-Alexa488, Thermo Fisher) was used to fluorescently label purified Pae87. A labelling reaction was set up by incubating the dye together with the protein in a 1:5 (protein:dye) ratio in 20 m*M* NaPiB pH 7.4, 100 m*M* NaCl for 1 h at room temperature. At this pH, N-terminal amino groups would be more reactive than lateral chain amino groups, and therefore the dye would preferentially be incorporated at the N-terminus. The reaction was stopped by adding a 10% volume of 1 *M* Tris–HCl pH 7.4. Free dye was then separated using a HiTrap Desalting FF 5 ml column on an ÄKTA start liquid-chromatography system with 20 m*M* NaPiB pH 7.4, 100 m*M* NaCl as the mobile phase. The much smaller molecule of the dye was retained for longer within the column, while the dyed protein eluted earlier. The degree of labelling of each elution sample was calculated by estimating the protein and dye concentrations, measuring *A*_280_ and *A*_495_, respectively, and applying the Lambert–Beer law with the respective molar extinction coefficients. *A*_280_ was corrected by a factor of 0.11 (corresponding to *A*_280_/*A*_495_ for the dye). Only samples with a labelling degree of ≃ 1 were used for experiments.

### 2.14. Statistical analyses

Statistical analyses were performed either in *R* or using *GraphPad InStat*. Unless otherwise stated, quantitative differences between experimental conditions were analyzed to determine whether these differences were statistically significant under the assumption of a significance level α = 0.05 using ANOVA. The *post hoc* tests of Tukey or Dunnett were further used for multiple comparisons. Unless otherwise stated, all results presented are mean ± standard deviation of at least three independent replicates.

## 3. Results

### 3.1. General analysis of the *Muramidase* family

**Figure 1:**
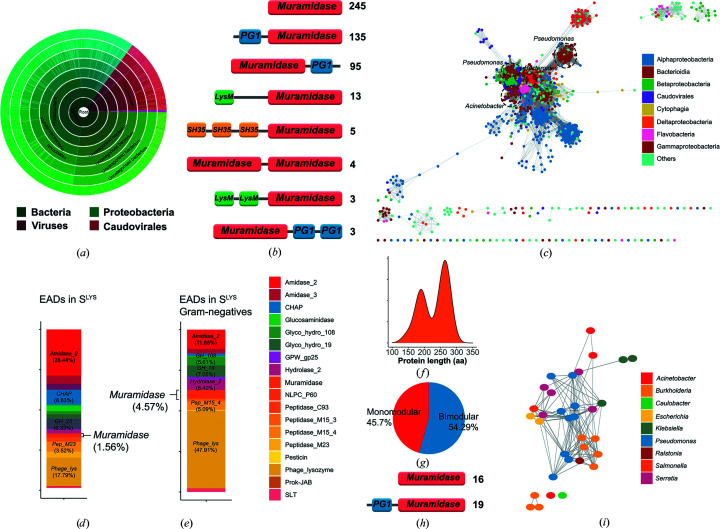
Overview of the *Muramidase* domain family. (*a*) Sunburst taxonomical representation of organisms bearing predicted *Muramidase* (PF11860)-containing proteins as taken from Pfam. (*b*) Main architectures found among *Muramidase*-containing proteins in Pfam. The numbers on the left are the number of representatives predicted to have such an architecture [PG1, blue boxes, PG_binding_1 (PF01471); SH35, orange boxes, SH3_5 (PF08460); LysM, green boxes, PF01476]. (*c*) SSN comprising the predicted *Muramidase*-bearing proteins in InterPro entry IPR024408 corresponding to the *Muramidase* family. (*d*, *e*) Distribution of catalytic domains in the phage lysin curated database 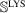 (*d*) or in the subset 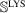 comprising only those lysins from phages that infect Gram-negative bacteria (*e*). (*f*) Length distribution of the *Muramidase*-containing lysins in 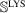 ( 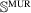 ). (*g*) Distribution of the number of predicted domains in 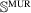 . (*h*) Architectures found in 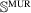 . (*i*) SSN of 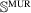 .

Pae87 contains a single predicted catalytic domain that occupies most of its full length (90.3% coverage) and belongs to the recently described *Muramidase* family (PF11860, Pfam *E*-value 1.1 × 10^−63^). The *Muramidase* family is thought to comprise *N*-acetylmuramidases (EC 3.2.1.17) which, as predicted by Pfam, are usually found in bacteria (mainly Proteobacteria) or bacteriophages from the Caudovirales family (Fig. 1 ▸ *a*). Lytic enzymes in Pfam containing a *Muram­idase* catalytic domain have architectures that range from a single *Muramidase* catalytic domain to *Muramidase* domains accompanied by different known CWBDs at either the N- or C-terminus (Fig. 1 ▸ *b*). A sequence-similarity network (SSN) constructed with the set of *Muramidase* family representatives (Fig. 1 ▸ *c*) contained in InterPro displays some similarity-based clustered groupings, such as those of proteins from *Pseudomonas* or some Alphaproteobacteria clusters, but in general it does not provide evidence of clear taxonomically relevant subfamilies. In a previously curated collection of phage lysin sequences named 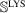 (Vázquez *et al.*, 2020 ▸), *Muramidase* domains accounted for 1.56% of the total predicted domains (Fig. 1 ▸ *d*), mostly represented in phages that infect Gram-negative bacteria (Fig. 1 ▸ *e*), among which the *Muramidase* family is not an uncommon catalytic domain. In the subset of 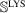 that comprises just those entries bearing at least a *Muramidase* catalytic domain, hereafter termed 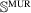 , the length distribution contains two subpopulations (Fig. 1 ▸ *f*) that can be related to the presence or absence of a CWBD, which is usually located at the N-terminus (Figs. 1 ▸ *g* and 1 ▸ *h*). 54.3% of the lysins in 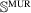 contained two predicted domains (*i.e.* bear both a *Muramidase* catalytic domain and a CWBD) while the rest are thought to be monomodular, as is Pae87 itself. The SSN of 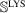 did not display a relevant taxonomical clustering (Fig. 1 ▸ *i*), although the sample size was on the low side to be able to draw generalized conclusions.

### 3.2. Three-dimensional structure of Pae87 and its substrate-binding region

While evidence has previously been provided for the antimicrobial activity of Pae87 (Vázquez, Blanco-Gañán *et al.*, 2021 ▸), in order to further elucidate the mechanism by which Pae87 interacts with susceptible bacterial cells we determined crystal structures of the protein with and without a bound peptidoglycan fragment.

The protein without and with PAO1 peptidoglycan was crystallized as described in Section 2. The best apoprotein crystal diffracted only to limited resolution and with somewhat streaky spots, but the best peptidoglycan-bound protein crystal diffracted X-rays to high resolution (Table 1 ▸) with cleaner spots. In the crystal packing, the peptidoglycan fragment contacts a neighbouring protein molecule; this may be the reason why the ligand-bound form crystallized in different conditions and in a different crystal form that diffracted X-rays to higher resolution. A structure-prediction search in *HHpred* (Zimmermann *et al.*, 2018 ▸) showed that AP3gp15, a *Burkholderia* AP3 phage endolysin that belongs to the *Muramidase* Pfam family, had the most similar sequence to Pae87 (50% identity with 183 amino acids in the alignment). Therefore, the apoprotein was solved by molecular replacement using AP3gp15 (Maciejewska *et al.*, 2017 ▸; PDB entry 5nm7) as a search model. The apo structure could only be refined to relatively high, but not unreasonable, *R*-factor values, while the peptidoglycan-bound Pae87 structure could be refined to more satisfactory values.

The crystallographic models of Pae87 comprise all 186 residues of the protein plus up to two residues from the N-terminal purification tag. The apo Pae87 and Pae87–peptidoglycan models have two and one molecules in the asymmetric unit, respectively. Molecule *B* of the apo structure is less well ordered than molecule *A*, as shown by the average temperature factors (68 versus 52 Å^2^, respectively). There is one amino acid with disallowed Ramachandran values in the apo structure: Gly32 in chain *B*. This residue has reasonable density and is near Leu76-Gly77 of chain *A*. Presumably, this contact pushes Gly32 into a somewhat unnatural conformation.

The root-mean-square deviations (r.m.s.d.s) between molecules *A* and *B* of the apo Pae87 structure and between molecules *A* or *B* of the apo structure and molecule *A* of the Pae87–peptidoglycan structure are 0.5 Å when C^α^ atoms are superposed. Since the structures are almost identical, we mainly describe the Pae87–peptidoglycan complex structure and only add a few details specific to the apo Pae87 structure.

**Figure 2:**
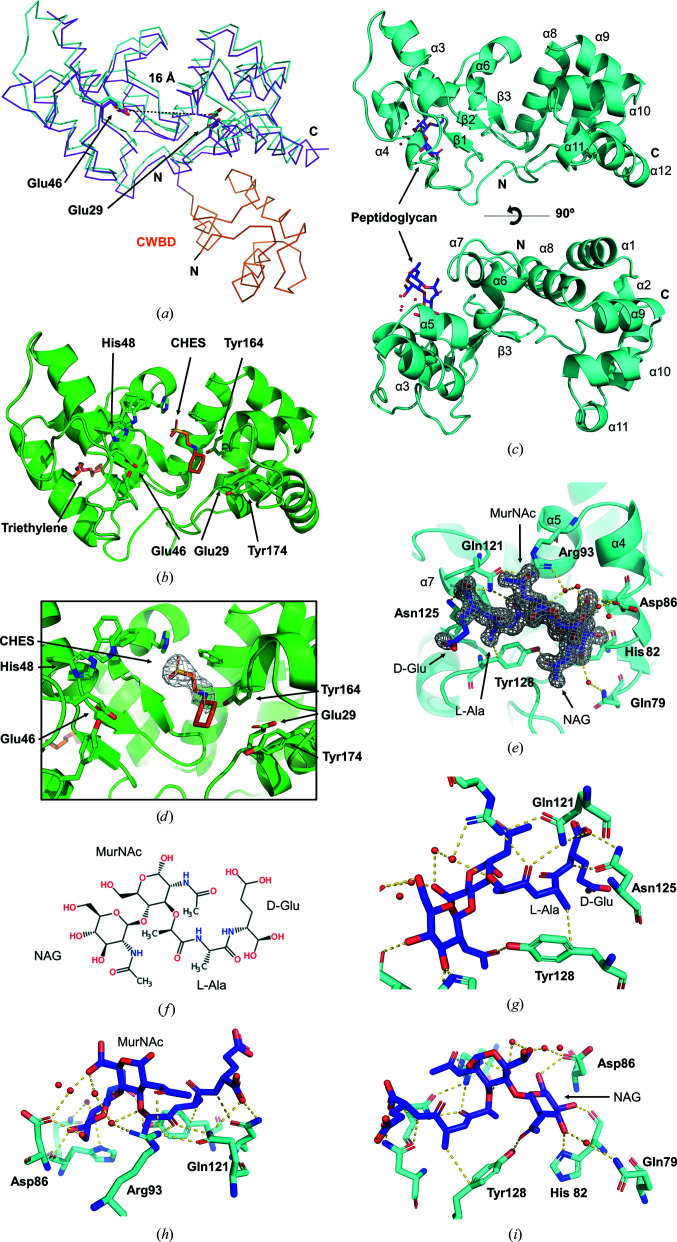
Pae87 structure. (*a*) Superimposition of the catalytic domains of Pae87 (cyan, PDB entry 7q4t) on AP3gp15 (magenta, PDB entry 5nm7), with the AP3gp15 CWBD shown in orange. The putative catalytic residues Glu29 and Glu46 from Pae87 and Glu101 and Glu118 from AP3g015 are shown in stick representation. (*b*) Ribbon representation of the apo Pae87 model coloured green (PDB entry 7q4s). A CHES molecule is depicted as orange sticks and is flanked by the putative catalytic amino acids (Glu29 and Glu46) and several aromatic residues in stick representation. A triethylene glycol fragment molecule (orange sticks) is present at the back of the protein, in the same place as the peptidoglycan in (*e*). (*c*) Side view (top) and top view (bottom) of the Pae87 protein model in ribbon representation (cyan). The peptidoglycan molecule is shown as violet sticks. (*d*) Close-up view of the active site depicted as in (*b*). The CHES molecule is represented with its 2*F*_o_ − *F*_c_ electron-density map contoured at 1σ (grey). (*e*) View of the peptidoglycan fragment depicted as in (*b*) with its 2*F*_o_ − *F*_c_ electron-density map contoured at 1σ (grey), the binding amino acids (cyan, stick representation) and the water molecules (red spheres) taking part in the hydrogen-bond network (yellow dashed lines). (*f*) Schematic representation in Fischer projection of the peptidoglycan fragment bound to Pae87. (*g*, *h*, *i*) Close-up views of the peptidoglycan fragment components (d-Gly and l-Ala, MurNAc and NAG, respectively) depicted as in (*c*).

The crystallographic structure of Pae87 is almost identical to the C-terminal catalytic domain of the endolysin AP3gp15 (r.m.s.d. value of 1.1 Å on 183 superposed C^α^ atoms). Pae87 lacks the N-terminal CWBD present in AP3gp15 (Fig. 2 ▸ *a*). The Pae87 structure shows the typical lysozyme-like α/β fold subdivided into two subdomains (Wohlkönig *et al.*, 2010 ▸). The α-lobe is composed of α-helices 1 and 2 (Glu5–Glu29) and α6–α12 (Gly109–Lys186). In the α-lobe subdomain, α2 is surrounded by the other α-helices. The other subdomain is traditionally known as the β-lobe because it contained a β-sheet in the first known endolysin structures (as it does in Pae87). In Pae87, the β-lobe is formed by β-strand 1 (Ile43–Glu46), α-helices 3, 4 and 5 (Arg47–Ser99) and β-strands 2 and 3 (Ala100–Met108). The three β-strands form a small antiparallel β-sheet (Fig. 2 ▸ *c*). Both subdomains form a cleft which contains the putative catalytic residues Glu29 and Glu46.

In the apoprotein crystal, a CHES molecule was bound in the cleft next to the catalytic amino acids (Figs. 2 ▸ *b* and 2 ▸ *d*). When Pae87 was crystallized together with the hydrolyzed peptido­glycan sacculi, we expected to see a fragment of it bound to the catalytic cleft. Surprisingly, the peptidoglycan fragment (comprised of linked NAG, MurNAc, l-alanine and d-glut­amic acid units; Fig. 2 ▸ *f*) was bound to α-helices 4, 5 and 7 on the opposite side to the putative catalytic pocket (Figs. 2 ▸ *c* and 2 ▸ *e*), in the same place in which a polyethylene glycol fragment was modelled in the apo Pae87 structure (Fig. 2 ▸ *b*). This fragment was mainly bound by a hydrogen-bond network formed by Gln79, His82 and Asp86 (α4), Arg93 (α5), Gln121, Asn125 and Tyr128 (α7) (Figs. 2 ▸ *g*–2 ▸ *i*). Tyr128 probably establishes a CH–π interaction with the l-Ala methyl group. The most important binding residues would be Arg93, Gln121, Asn125 and Tyr128, because they each make more than one bond to the ligand.

**Figure 3:**
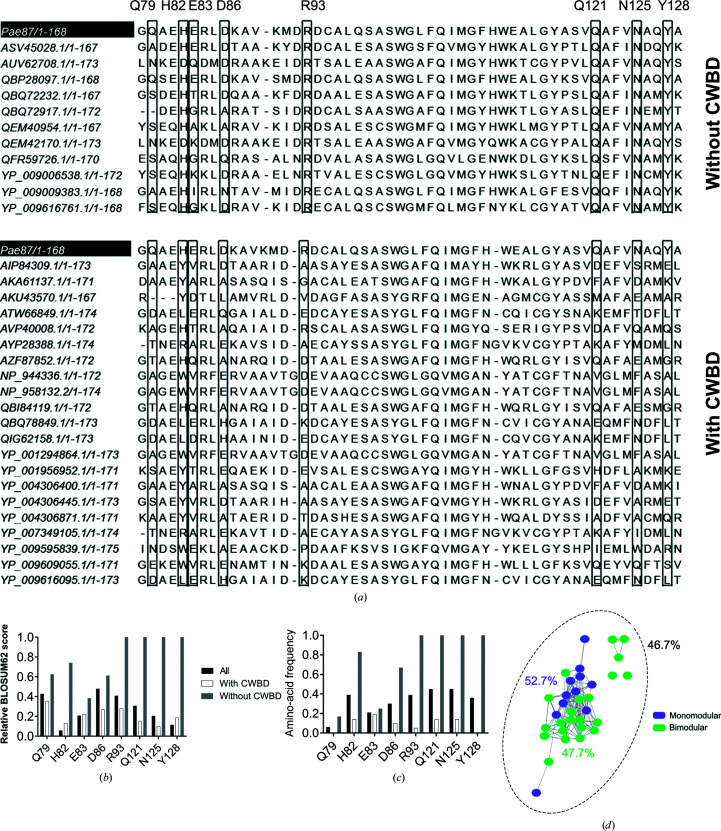
Multiple sequence alignments (MSAs) of the peptidoglycan-binding site of lysins in 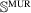 . (*a*) MSA of the peptidoglycan-binding site of *Muramidase* family lysins grouped by the presence or absence of an N-terminal CWBD. The residue coordinates indicated at the top are those of Pae87. (*b*, *c*) Residue-conservation metrics for the residues putatively involved in contacts with peptidoglycan at the peptidoglycan-binding site according to the Pae87 3D model. The relative BLOSUM62 score is shown in (*b*) and the relative frequency of the Pae87 residue across the MSAs in each respective position is shown in (*c*). (*d*) SSN of the 33 sequences of the MSAs distinguished by the presence or absence of a CWBD. Percentages are average identities for each group. The entries AKU43570.1 and YP_009595839.1 were reclassified as CWBD-bearing lysins since they contained an unidentified N-terminal end that could probably be an as yet undescribed CWBD.

A multiple sequence alignment (MSA) analysis of the amino acids composing the peptidoglycan-binding site using 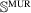 (removing two low-coverage examples: YP_009639957.1 and YP_337984) revealed that the residues relevant for peptidoglycan binding were much more conserved in those lysins bearing a single *Muramidase* catalytic domain than in those which had an additional CWBD (Fig. 3 ▸ *a*). Specifically, in the case of Tyr128 and Arg93, together with Gln121 and Asn125, both the amino-acid frequency and the relative BLOSUM62 score (which is a similarity metric) were close to the maximum value (1.0) for the non-CWBD-bearing entries (Figs. 3 ▸ *b* and 3 ▸ *c*). For the bimodular lysins of the *Muramidase* family, the scores were below 0.4 and 0.2 for Tyr128 and Arg93, respectively. The SSN in Fig. 3 ▸(*d*) shows that no sequence-identity bias was introduced by the classification since the average identity percentage both within each sub­group and in the whole collection of sequences was around 46–52% and there was no apparent clustering.

### 3.3. Analysis of the Pae87 catalytic centre

**Figure 4:**
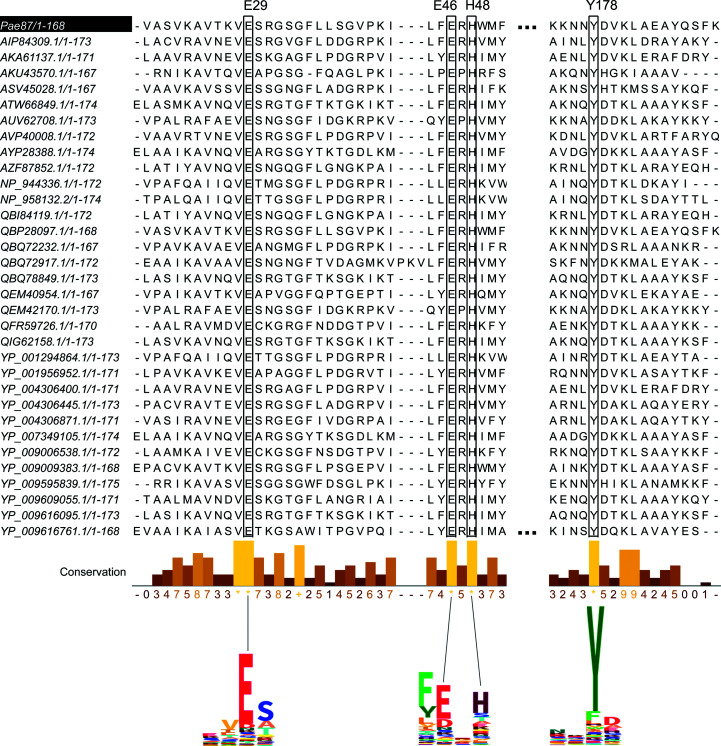
MSA of *Muramidase* family domains in 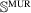 showing the conservation of residues facing the catalytic pocket. Selected segments of the Pfam HMM logo of the family are displayed at the bottom. Relevant positions are connected to their respective columns in the MSA.

A single glutamic acid has been pointed out to be involved in catalysis in AP3gp15 (Maciejewska *et al.*, 2017 ▸). A corresponding Glu residue is also conserved in the Pae87 structure (Glu29; Fig. 4 ▸) and in all of the 32 *Muramidase* sequence examples in the curated 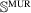 . Glu29 is located within a deep cleft between the subdomains of the Pae87 structure (Fig. 2 ▸ *a*), and thus this is considered to be the catalytic site. This glutamic acid residue is conserved in many endolysins, such as AP3gp15 (PDB entry 5nm7; Maciejewska *et al.*, 2017 ▸), hen egg-white lysozyme (HEWL; PDB entry 4hpi; Ogata *et al.*, 2013 ▸) and the peptidoglycan hydrolase Auto (PDB entry 3fi7; Bublitz *et al.*, 2009 ▸). Most lysozymes have a catalytic site formed by two residues, a general acid and a general base, which are normally acidic amino acids (Glu or Asp). The C-terminal region of the central α-helix (α2) of the lysozyme-like α/β fold typically contains a conserved catalytic glutamate, but the general base is not well conserved and does not exist in some lysozymes. In HEWL it is located 5 Å away at the other side of the cleft, and in the peptidoglycan hydrolase Auto the general base is 14 Å away in the antiparallel β-sheet (Bublitz *et al.*, 2009 ▸).

Recently published results regarding the bimodular lysin LysT84, which contains an N-terminal PG_binding_1 (PF01471) CWBD plus a *Muramidase* enzymatically active domain, as AP3gp15 does, have pointed out the involvement of a second Glu residue in the catalytic activity (Love *et al.*, 2022 ▸). Indeed, this second Glu residue (Glu46 in Pae87) was found to be perfectly conserved across all of the *Muramidase* examples considered in this work (Fig. 4 ▸). This perfect conservation strongly suggests that it plays a relevant role, possibly as the general base, located at a distance of 16 Å from Glu29 (Fig. 2 ▸ *a*), in a similar fashion as described for the Auto peptidoglycan hydrolase.

Other conserved residues facing the catalytic cleft were His48 and Tyr174, which therefore could also play a significant role in the integrity and function of the catalytic pocket. The aforementioned residues also presented high conservation levels within the Pfam hidden Markov models (HMMs) of the family, judging by the HMM logos (Fig. 4 ▸), strengthening their presumptive functional role. In the apo Pae87 structure, electron density for a CHES buffer molecule was found inside the putative catalytic cleft between the putative catalytic glutamates (Figs. 2 ▸ *b* and 2 ▸ *d*). Buffer molecules have also been found in the catalytic clefts of other enzymes, such as the lytic CHAP_K_ domain of the endolysin LysK from *Staphylococcus* bacteriophage K or chitinase C (ChiC) from *Streptomyces griseus* HUT6037 (Sanz-Gaitero *et al.*, 2014 ▸; Kezuka *et al.*, 2006 ▸). They may mimic the enzymatic substrate or product, and thus their position reinforces the idea that Glu29 and Glu46 possess catalytic activity in Pae87.

**Figure 5:**
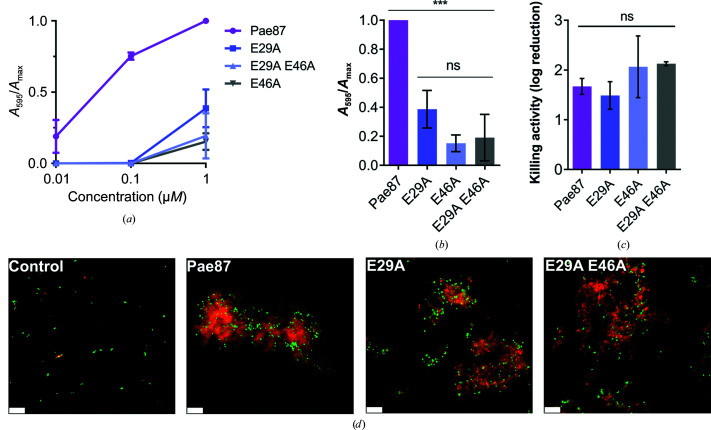
Activities of wild-type Pae87 and of mutants in which conserved catalytic site glutamic acid residues were mutated. (*a*) Muralytic activity of Pae87 and its mutants on RBB-labelled purified PAO1 cell walls at different concentrations. (*b*) Comparison of muralytic activities at the maximum concentration tested (1 µ*M*). (*c*) Bactericidal activity of 10 µ*M* Pae87 and its mutants against bacterial suspensions of PAO1 (2 h, 37°C). (*d*) Fluorescence microscopy of some of the treated suspensions in (*c*) dyed with SYTO9 and propidium iodide. White bars at the lower left corner indicate the scale (10 µm). One-way ANOVA was used in (*b*) and (*c*) followed by a Tukey post-test to perform an all-against-all multiple comparison (ns, non-significant difference; ***, *p* ≤ 0.01).

A mutational analysis was conducted to confirm the implications of both Glu29 and Glu46. Pae87 single mutants (E29A and E46A) and a double mutant (E29A/E46A) were constructed and their muralytic and bactericidal activities were tested (Fig. 5 ▸). Both residues, Glu29 and Glu46, were deemed to be relevant for the catalytic degradation of PAO1 peptidoglycan since all of the mutants displayed a remarkable decrease in their cell-wall solubilization ability when compared with wild-type Pae87 (Figs. 5 ▸ *a* and 5 ▸ *b*). At the maximum nonsaturating concentration (0.1 µ*M*), Pae87 retained about 75% of its maximum detected activity, while the mutants displayed no peptidoglycan solubilization activity. On the other hand, there were no significant differences in the observed bactericidal activity against PAO1 between Pae87 and its mutants or in the fluorescence microscopy images (Figs. 5 ▸ *c* and 5 ▸ *d*).

**Figure 6:**
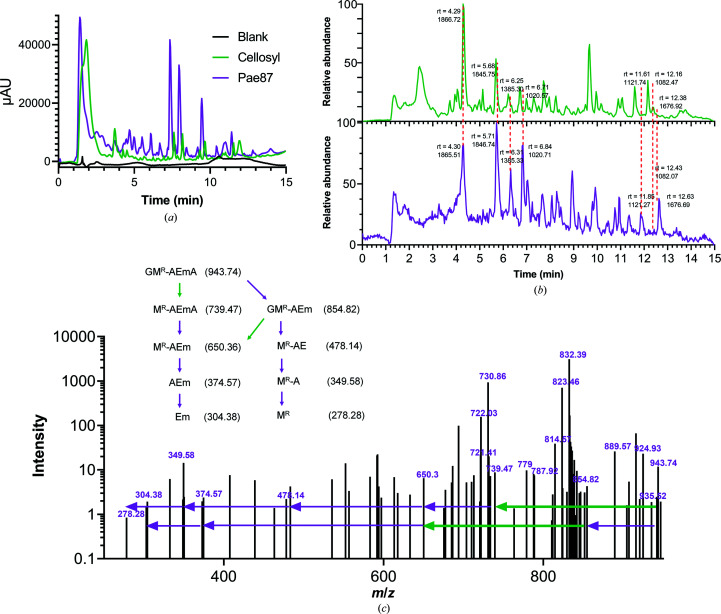
Analysis of the degradation products of Pae87 activity on *P. aeruginosa* PAO1 peptidoglycan. (*a*) UV (204 nm) chromatograms of PAO1 peptidoglycan solubilized with either cellosyl or Pae87 and of the soluble fraction of an untreated sample. (*b*) Liquid chromatography–mass spectrometry chromatograms of the degradation products of cellosyl (green) and Pae87 (purple) activities on PAO1 peptidoglycan. The retention time (rt) and a representative monoisotopic mass value are shown for selected peaks. (*c*) CID spectrum of the rt = 4.30 min peak of the Pae87 peptidoglycan degradation. Selected *m*/*z* values are displayed. The dissociation of a GM^R^-AEmA fragment is presented. G, nonreduced NAG; M^R^, reduced MurNAc; A, alanine; E, glutamic acid; m, *meso*-diaminopimelic acid.

Regarding the catalytic activity of Pae87, the disaccharide found in the soluble product bound to the crystallized protein (NAG-MurNAc rather than MurNAc-NAG) already indicates lysozyme activity, as described in the literature for this family. Analysis of the degradation products by RP-HPLC-MS firstly showed that both Pae87 and the positive control (the lysozyme cellosyl; Rau *et al.*, 2001 ▸) mobilized an array of soluble compounds, in contrast to the untreated blank (Fig. 6 ▸ *a*). When comparing the mass-spectrometry chromatograms of cellosyl and Pae87, a coincidental pattern for the main degradation peaks was found (Fig. 6 ▸ *b*). This observation supports the catalytic nature of Pae87 as a muramidase. Moreover, the CID spectrum of one of the main peaks of Pae87-degraded peptido­glycan (that at 4.3 min) presents peaks that are compatible with the loss of a nonreduced NAG (−203.078 mass units) from a NAG-MurNAc-Ala-Glu-mDAP-Ala or a NAG-MurNAc-Ala-Glu-mDAP fragment (Fig. 6 ▸ *c*). Conversely, there is no evidence that is coherently compatible with the loss of a reduced NAG (−223.106).

### 3.4. The noncatalytic activity of Pae87 and P87

**Figure 7:**
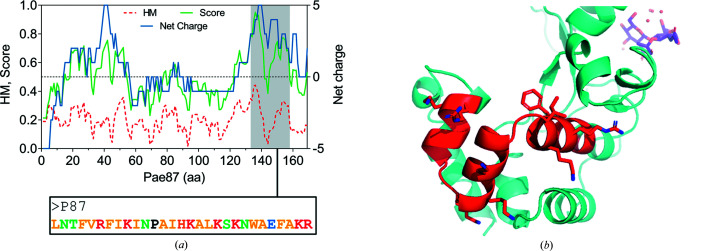
Definition and localization of the P87 peptide within Pae87. (*a*) Physicochemical profile of Pae87, depicting the HM and net charge in each of the 11-amino-acid windows of the protein, as well as the score used to measure both of these properties. The P87 sequence is also shown colour-coded with respect to the properties of each residue (yellow, nonpolar; green, polar without charge; red, positively charged; black, proline; blue, negatively charged). (*b*) 3D model of Pae87 with peptide P87 highlighted in red.

The hypothesis that Pae87 displays a noncatalytic bacteri­cidal activity has previously been proposed (Vázquez, Blanco-Gañán *et al.*, 2021 ▸), and it is consistent with the results presented in Fig. 5 ▸. A closer examination of the physicochemical profile of Pae87 allowed us to postulate a specific 29-amino-acid peptide, named P87, which would make up a C-terminal AMP-like region (Fig. 7 ▸). The definition of P87 was based on the concurrent maximization of net charge and HM that takes place in the coordinates of Pae87. To measure these properties simultaneously, a score variable was calculated as explained in Section 2. The P87 sequence contains seven positively charged Arg or Lys residues (and a negatively charged Glu), interspersed with mostly nonpolar residues. This structure, together with the fact that it forms three α-helices within the Pae87 structure (Fig. 7 ▸), suggests that it may form amphipathic helices with membrane-interaction potential.

**Figure 8:**
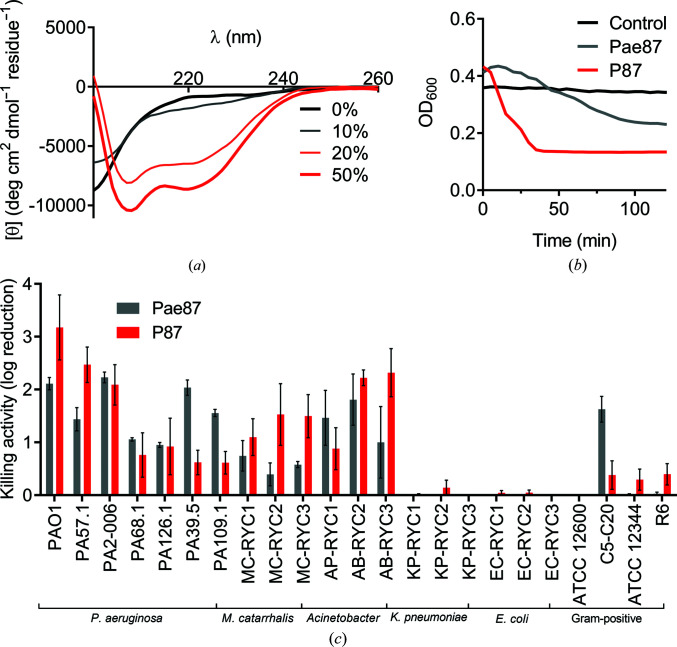
Antimicrobial activity of P87 peptide. (*a*) Far-UV circular-dichroism spectra of 20 µ*M* P87 in 20 m*M* NaPiB pH 6.0 with 100 m*M* NaCl and different (*v*/*v*) concentrations of TFE (as indicated in the legend). (*b*) Turbidity decrease assay of a 10 µ*M* P87 or Pae87 treatment of PAO1 cell suspensions. (*c*) Bactericidal activity range of 10 µ*M* P87. The Pae87 bactericidal range from Vázquez, Blanco-Gañán *et al.* (2021 ▸) is also shown for comparison.

The antimicrobial properties of the synthetic peptide P87 were analyzed. Circular-dichroism spectra in the presence of increasing concentrations of TFE provided evidence for the ability of P87 to form amphipathic helices in the presence of biological membranes (Fig. 8 ▸ *a*). P87 presented a disordered conformation in aqueous solution; however, beginning at 20% TFE it started to shift towards an α-helical conformation, as shown by the typical peaks at approximately 222 and 208 nm. An acute lytic effect of P87 on *P. aeruginosa* PAO1 was observed, in contrast to the lack of generalized lysis when using the full Pae87 protein (Fig. 8 ▸ *b*). An OD_600_ peak at *t*_0_ is observed for both Pae87 and P87 above the turbidity value of the control. This has been noted previously when testing surface-active proteins on intact bacterial suspensions (Vázquez, Blanco-Gañán *et al.*, 2021 ▸), and we speculate that it is due to the cell aggregation induced by the treatment. The subsequent decrease in turbidity should then be due to cell disintegration (which is much more acute in the case of the highly lytic peptide) and/or the formation of larger cell-debris aggregates that leave regions of the bottom of the well free of cells, thus potentially leading to a lower OD value. P87 was also able to kill PAO1 and other bacteria with a similar range and magnitude to Pae87 (Fig. 8 ▸ *c*). The antimicrobial capacity of P87 is thus considered to be proven, and therefore a role in the Pae87–bacterial surface interaction can be proposed with supporting evidence.

### 3.5. Antimicrobial mechanism of Pae87 and P87

**Figure 9:**
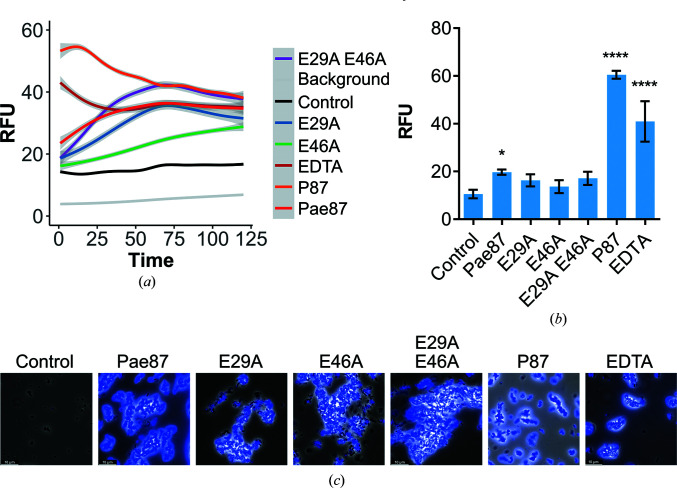
OM permeabilization assays with the fluorescent probe NPN. (*a*) Generalized additive model estimation of the average tendencies of the NPN fluorescence kinetics (the excitation wavelength was 350 nm and emission was recorded at 420 nm). Estimation was based on three independent replicates; the mean estimation ± 95% confidence interval (shaded grey) is shown for each experimental condition. (*b*) Comparison of the average NPN signal (minus background fluorescence) after 5 min incubation. The mean ± standard deviation of three independent replicates is shown; a one-way ANOVA test with a Dunnett post-test was applied to statistically compare each condition with the control (untreated cells in the presence of the probe). *, *p* ≤ 0.05; ****, *p* ≤ 0.0001. (*c*) Fluorescence microscopy observation of each experimental condition after 2 h incubation. Superimpositions of phase-contrast images with blue fluorescence signal observed with an A filter cube (excitation bandpass 340–380 nm) are shown. RFU, fluorescence units relative to the maximum value achieved during the assay.

Since the OM has traditionally been labelled as the main obstacle when attacking Gram-negative bacteria from without, a fluorescent probe was used to detect OM permeabilization induced by Pae87 and its derivatives (*i.e.* the noncatalytic mutants and the antimicrobial peptide P87). NPN is a hydrophobic molecule that fluoresces when it reaches the phospholipid layer of the inner membrane upon OM permeabilization. Typically, NPN uptake kinetics are recorded with short incubation times (with a maximum of 10 min and a minimum of 3 min; Loh *et al.*, 1984 ▸; Helander & Mattila-Sandholm, 2000 ▸). However, we extended the incubation period to 2 h due to the increasing fluorescence values of Pae87 kinetics over such a time (Fig. 9 ▸ *a*). In the average kinetic estimations shown in Fig. 9 ▸(*a*), two different tendencies can be observed: the ‘canonical’ OM permeabilization peak induced by P87 in the first minutes after peptide addition and the slow but steady increase in fluorescence for Pae87 and its mutants. For the sake of statistical comparison, the average values of the NPN fluorescence reached 5 min after reagent addition were considered (Fig. 9 ▸ *b*). Over such a short period the NPN fluorescence of Pae87 and its noncatalytic mutants is still low, although for Pae87 it is significantly different from the control. The fluorescence induced by P87 was greater than that obtained for the EDTA positive control. Fluorescence microscopy images at the end of incubation visually confirmed the damage to the OM for all of the compounds tested (Fig. 9 ▸ *a*).

**Figure 10:**
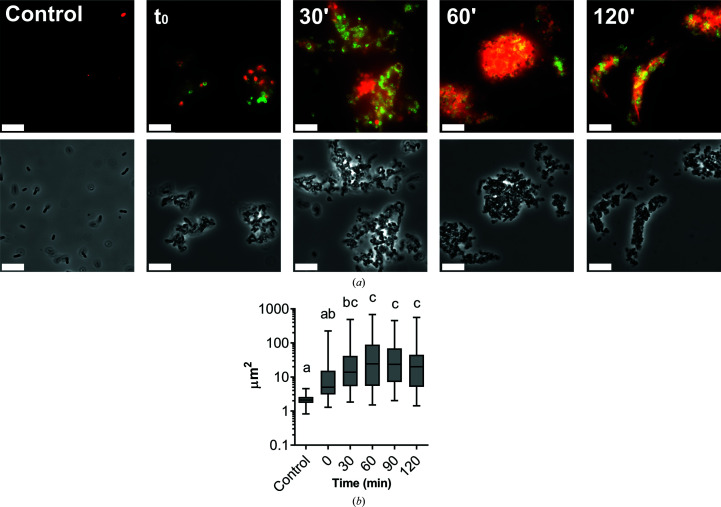
Microscopic observation of a PAO1 suspension treated with Pae87 over time. (*a*) Fluorescence and phase-contrast images representative of the observations made during 2 h of incubation (37°C, 20 m*M* NaPiB pH 6.0, 150 m*M* NaCl) of a PAO1 suspension (∼10^8^ CFU ml^−1^) treated with 10 µ*M* Alexa488-labelled Pae87 and stained with PI. White bars indicate 10 µm. (*b*) Time-wise distributions of cells or aggregate areas estimated using the *LAS X* microscopy image-analysis software for at least ten frames per time point. A one-way ANOVA followed by a Tukey’s post-test was applied for multiple comparisons. Distributions marked with different letters are significantly different from each other, while no significant differences were found between those indicated with the same letter.

A fluorescent version of Pae87 labelled with Alexa488 was used in combination with PI, a DNA-intercalating fluorescent probe. In this way, the temporal localization of Pae87 and its effect on a suspension of PAO1 cells were traced (Fig. 10 ▸). At *t*_0_ Pae87 had already begun binding to the bacterial surface, as green fluorescence rims were observed around the *P. aeruginosa* cells (Fig. 10 ▸ *a*). Over time, the Pae87 molecules bound to the cell walls promoted increasing aggregation among close bacteria. According to visual estimations of the areas of these aggregates, their maximum size was reached after approximately 30–60 min under the assay conditions (Fig. 10 ▸ *b*). Also, at *t*_0_ a discrete intracellular spot of red fluorescence (PI) was observed. Subsequently, the PI fluorescence underwent a gradual diffusion concomitant to the aggregation. At 60 min of incubation, red fluorescence appeared as a halo around the aggregates, with a distorted bacterial morphology.

**Figure 11:**
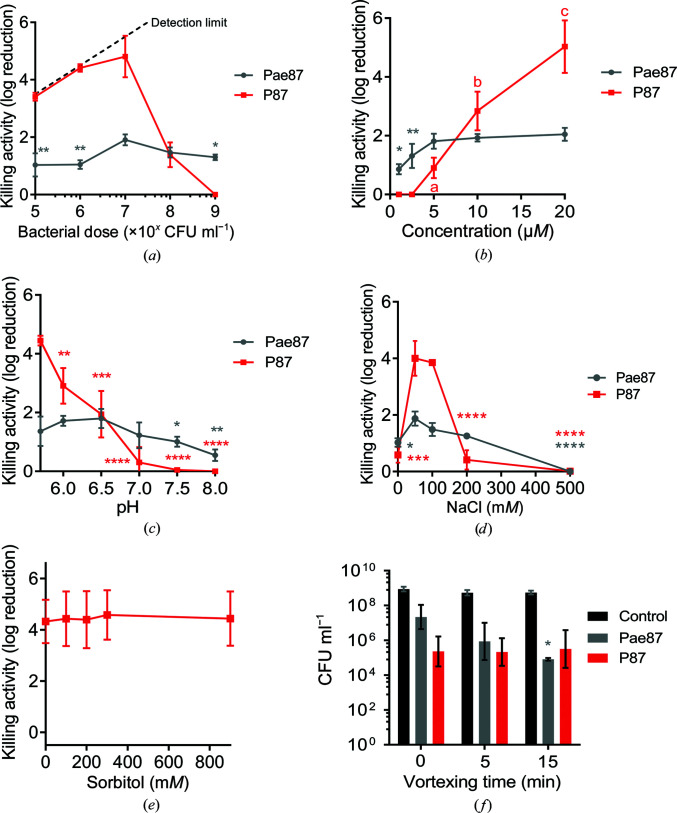
Parameters of Pae87 and P87 bactericidal activity on *P. aeruginosa* PAO1. (*a*) Enzyme:bacteria or peptide:bacteria stoichiometry. (*b*) Dose–response curves. (*c*) Variation of killing activity with pH. (*d*) Variation of killing activity with ionic strength. (*e*) Variation of killing activity with the concentration of a non-ionic osmolyte (sorbitol), maintaining a fixed concentration of 50 m*M* NaCl. (*f*) Viable counts after 0, 5 or 15 min of vortexing. One-way ANOVA with a Dunnett post-test was applied for comparison with the control or the highest value condition: (*a*) 10^7^ CFU ml^−1^, (*b*) 20 µ*M*, (*c*) 6.5, (*d*) 50 or 150 m*M*, (*e*) 0 m*M* sorbitol and (*f*) the corresponding viable count without vortex treatment. Unless otherwise stated, the incubation conditions were 20 m*M* NaPiB pH 6.0, 150 m*M* NaCl, ∼10^8^ CFU ml^−1^ PAO1, 10 µ*M* of the bactericidal compound, 37°C, 2 h. Asterisks indicate *p*-values of significant comparisons (*, *p* ≤ 0.05; **, *p* ≤ 0.01; ***, *p* ≤ 0.001; ****, *p* ≤ 0.0001); nonsignificant comparisons are not indicated. When marked by letters, an all-against-all multiple comparison was applied with ANOVA plus a Tukey post-test. Different letters indicate significantly different results.

Several incubation conditions were examined to check their importance in Pae87 and P87 killing activity (Fig. 11 ▸). The peptide:bacteria stoichiometry was critical for the killing efficacy of the P87 peptide and, to a lesser extent, of Pae87 (Fig. 11 ▸ *a*). In both cases the optimal ratio was achieved at around 10^7^ CFU ml^−1^ for 10 µ*M* Pae87 or P87, which roughly corresponds to some 10^10^ molecules per cell. The dose–response curve of Pae87 quickly saturated (Fig. 11 ▸ *b*), as has been shown before (Vázquez, Blanco-Gañán *et al.*, 2021 ▸). This was speculated to be due to the entrapment of Pae87 molecules within the aggregates. P87 presents a more canonical curve, in line with the effect of the peptide:bacteria ratio, with an increasing activity with higher concentrations (up to a 5 log increase in killing at the maximum concentration tested).

pH did not seem to be a major determinant of Pae87 activity (Fig. 11 ▸ *c*), although the best killing numbers were obtained at mildly acidic pH, as for P87. Ionic strength did not have a remarkable impact at near-physiological concentrations (50–200 m*M* NaCl) for Pae87, but P87 only displayed a relevant killing activity between 50 and 150 m*M* (Fig. 11 ▸ *d*). It is possible that a slight salt concentration might be necessary for proper P87 solubility (in fact, the *Aggrescan* server detects an N-terminal hotspot for aggregation; de Groot *et al.*, 2012 ▸) but that higher concentrations might shield charged residues. This latter hypothesis is supported by the results in Fig. 11 ▸(*e*), in which the activity of 10 µ*M* P87 was tested in the presence of different concentrations of a non-ionic osmolyte (sorbitol). In these results, the killing activity of the peptide was not affected by increasing concentrations of the non-ionic solute. Finally, PAO1 suspensions that had been treated for 2 h were subjected to different vortexing treatments before plating to check whether the observed aggregative effect was underestimating the viable cell counts (Fig. 11 ▸ *f*). The vortex treatment would disintegrate the aggregates and thus release viable cells that, grouped within the aggregates, might have produced a single colony. The results in Fig. 11 ▸(*f*), however, suggest that this is not the case, *i.e.* the observed decrease in viable cell counts cannot be solely attributed to mere aggregation, and thus a bactericidal mechanism must be occurring. This is because no significant differences were found in the viable counts after vortexing with respect to the nonvortexed samples, except for the samples treated with Pae87 and vortexed for 15 min. In this latter case, the cell count in fact decreased over the control without vortexing.

## 4. Discussion

Knowledge of the structure and function of phage lysins is a crucial step towards a deeper understanding of how lysins work and their application as engineered antimicrobials. Pae87 has previously been identified and confirmed to be an intrinsically active lysin against a range of Gram-negative pathogens. In this work, this activity has been further investigated in relation to the protein structure to try to unveil the way that Pae87 works as an exogenous antimicrobial.

The structure of Pae87 shows that it is a one-domain protein with the typical α/β lysozyme fold. In addition, three α-helices are inserted between the β-strands of the β-sheet, together forming the β-lobe. A comparable structure was only found in AP3gp15, a *Burkholderia* phage lysin. A peptidoglycan fragment was bound to this region of the β-lobe in the Pae87–peptido­glycan crystal structure. Interestingly, the residues predicted to be responsible for the binding of the peptido­glycan fragment were more conserved in representatives from the *Muramidase* family that lack an additional CWBD than in those that possess one (such as AP3gp15). From these results, the existence of a cell-wall-binding site within the catalytic domain can be proposed. In the absence of a CWBD, this region may be used as a binding site by Pae87 and the other related lysins that contain it, perhaps performing a function in the endogenous lytic activity. In fact, most carbo­hydrate enzymes known to date possess a CWBD that enables them to approach their substrate, since polysaccharidic substrates are insoluble and thus cannot diffuse into the catalytic centre (Guillén *et al.*, 2010 ▸). The observations presented in this work imply that the hypothesis that Gram-negative phage lysins lack or do not need a cell-wall-binding function (Ghose & Euler, 2020 ▸; Vázquez, García *et al.*, 2021 ▸) should perhaps be revised once sufficient evidence has been gathered on (i) whether these internal regions within the catalytic domain function as true CWBDs and (ii) how widespread this trait is. To our knowledge, there are few examples in the literature of cell-wall-binding sites contained within the catalytic domain. For example, *Bacillus* lysin PlyG contains a region located within the NAM-amidase catalytic domain that specifically recognizes *B. anthracis* spores (Yang *et al.*, 2012 ▸). From a functional point of view, this is hardly a comparable case, since the secondary substrate-binding site of PlyG seems to have evolved to recognize a chemically distinct form of the target bacterium that cannot be recognized by the canonical CWBD. A binding region far from the active site has also been proposed at the C-terminal lobe of T4 lysozyme (Grütter & Matthews, 1982 ▸; Kuroki *et al.*, 1993 ▸), and in this case it was proposed to bind the peptide moiety of the peptidoglycan, thus providing some specificity to the lytic activity. In contrast, for Pae87 and its close relatives it could be argued that a region with affinity for peptidoglycan has arisen to take over the function of the lacking CWBD, given the differential conservation of the predicted key residues for binding.

Unlike the binding motif, the Pae87 residues of the catalytic cleft are conserved in AP3gp15 (Fig. 2 ▸ *a*). Here, the most important residues are Glu29 and Glu46, which are responsible for the catalytic activity. Glu29 may correspond to the general acid of other enzymes of the lysozyme family (Wohlkönig *et al.*, 2010 ▸). An equivalent to Glu46, the presumed general base, is only found in some lysozymes, such as the peptidoglycan hydrolase Auto (Bublitz *et al.*, 2009 ▸) and AP3gp15, although the function of the equivalent Glu46 in the highly homologous AP3gp15 was not studied and in fact it was not pointed out as a possible catalytic residue (Maciejewska *et al.*, 2017 ▸). The conserved residues in the MSAs presented in this work have allowed the identification of this glutamic acid and we also showed that an alanine mutant of Glu46 abolishes lysozyme activity in Pae87, therefore confirming the catalytic function of both Glu29 and Glu46. This is further supported by the recent results of Love *et al.* (2022 ▸), which show that a pair of Glu residues are critical for catalysis in the *Muramidase* family member bimodular LysT84. However, the distance between the Pae87 glutamates is 16 Å, which is longer than the typical distance in inverting glycoside hydrolases (Davies & Henrissat, 1995 ▸). For context, the typical O⋯O inter-carboxylic distance has been recorded to be 8.5 ± 2.0 Å on average in inverting β-glycosidases, while it is shorter (4.8 ± 0.3 or 6.4 ± 0.6 Å) in β-glycosidases that use the retaining mechanism (Mhlongo *et al.*, 2014 ▸). A putative conclusion from these observations is that the hydrolysis mechanism of Pae87 may operate with net inversion of the anomeric configuration. In fact, Bublitz and coworkers proposed that Auto could move to a closed conformation when it binds to its substrate and thus follow the inverting mechanism of glycoside hydrolysis. The same conformational readjustment and mechanism could occur in Pae87. This hypothesis has been tested for LysT84 by molecular-dynamics simulations, the results of which were compatible with a minimum allowed Glu–Glu distance of around 9–10 Å, which is within the range of inverting glyco­sidases (Love *et al.*, 2022 ▸). While mutating Glu29 and Glu46 dramatically diminished the muralytic efficacy of Pae87, the three mutants (including the E29/E46 double mutant) also retained some residual activity at the maximum concentration tested (less than 50% of that of the wild-type enzyme), suggesting that other residues may be involved in catalysis (perhaps such conserved residues as His48 and/or Tyr174), or rather that some residues in the catalytic cleft may take over the function of the mutated amino acid to some degree. Previous analyses of the precise catalytic activity of lysins from the *Muramidase* family have all concluded that they actually possess a muramidase or lysozyme activity that breaks the glycan chain of the peptidoglycan on the reducing side of MurNAc (Rodríguez-Rubio *et al.*, 2016 ▸; Maciejewska *et al.*, 2017 ▸). Our results also fit with this proposal. To begin with, correspondence was found between the main degradation peaks detected by RP-HPLC-MS when treating *P. aeruginosa* peptidoglycan with cellosyl, a known lysozyme, and with Pae87. Some of the coincidental main peaks can easily be assigned to peptidoglycan-degradation products. For example, the main peaks with a monoisotopic *m*/*z* of ∼1865 or ∼1845 are consistent with fragments containing two disaccharides [NAG-(reduced MurNAc)] cross-linked by two tetrapeptides (Ala-Glu-mDAP-Ala). The difference in mass (1865 versus 1845) corresponds to loss of a water molecule, probably from one of the reduced MurNAc residues. The CID results are definitive proof of a muramidase activity, which leaves a MurNAc end susceptible to reduction (conversely, NAG is susceptible to reduction when the glycan strand is degraded by a glucosaminidase activity; Eckert *et al.*, 2006 ▸; Rodríguez-Rubio *et al.*, 2016 ▸).

On the other hand, the lack of a difference in bactericidal activity between Pae87 and its mutants suggests that the membrane activity is the major determinant of antimicrobial potential in Pae87, rather than the catalytic activity, as pointed out for other intrinsically active lysins (Ibrahim *et al.*, 1996 ▸). In this work, a specific AMP-like C-terminal region (P87) with intrinsic membrane-permeabilizing and bactericidal activity has been identified as the part of the enzyme most probably responsible for the aforementioned effect. The crystal structure confirmed that P87 is located on the surface of the protein, supporting the hypothesis that it would be able to directly interact with membranes. Moreover, P87 by itself had an OM permeabilizing activity, as demonstrated by the NPN uptake assay. Nonpolar residues of P87 are mostly buried within Pae87, but not in all cases. For example, Ile164 or even Phe161 are especially exposed (Fig. 7 ▸ *b*). The Lys and Arg residues of P87, on the other hand, are located on the outer surface of the protein and are therefore available for electrostatic interaction with negatively charged elements of the bacterial surface (namely, the phosphate groups of the lipopolysaccharide). Based on the results in this work, including the microscopy images presented in Fig. 10 ▸, a mechanism for Pae87 activity from without is proposed: (i) the P87 region of the enzyme would bind to the OM, coating the bacterial surface and then causing the aggregation of adjacent cells, (ii) the membrane-permeabilizing action would then act, perhaps together with the peptidoglycan hydrolysis activity, to disrupt the cell wall and (iii) the leakage of intracellular components and cell death takes place without provoking full disintegration of the bacteria (‘lysis’), but rather keeping the cell debris tightly bound in compact aggregates. The decreases in viability after vortexing presented in Fig. 11 ▸ are in agreement with the proposal that the aggregates comprise damaged cells (not relatively intact ones) and thus mechanical shaking increased the apparent killing by definitely harming these already damaged bacteria. This ‘death without lysis’ could be beneficial from the point of view of *in vivo* therapy, since it would prevent the dissemination of pro-inflammatory factors. Also, bacterial aggregates have previously been shown to be better cleared by the immune system (Ribes *et al.*, 2013 ▸; Roig-Molina *et al.*, 2020 ▸). However, the true potential of this kind of antimicrobial agent should be tested *in vivo* to clarify its possible benefits. This proposed mechanism applies to Pae87 when exogenously used as an antibacterial agent. On the other hand, and judging by the previously reported analysis of the phage JG004 lytic cassette (Vázquez, Blanco-Gañán *et al.*, 2021 ▸), Pae87 is thought to behave as a canonical endolysin during the infective cycle of the phage, *i.e.* Pae87 should be exported to the periplasm through holin-formed pores at the late infection stage and thus exert its peptidoglycan-degradation activity. Once the peptidoglycan is sufficiently degraded, the putative two-component spanin system should assemble to bring together the inner and outer membrane and create larger pores through which the viral progeny is released, also causing effective lysis of the bacterial host. Within this rather classical framework, the sole function of Pae87 would be to degrade the peptidoglycan. However, this does not exhaustively account for many of the structural–functional features that have beeen uncovered in this work. For example, it is still unclear why many Gram-negative lysins have evolved an AMP-like motif (Vázquez, García *et al.*, 2021 ▸). We may speculate that the increased affinity towards the bacterial surface provided by the cationic patch may (i) be beneficial for approaching the insoluble substrate and/or (ii) assist in the permeabilization of the membrane(s) towards effective lysis. In addition, the binding to the cell surface may also be relevant to maintain lysins tightly bound to cell debris, thus preventing a potentially toxic effect in the neighbouring cells. This hypothesis has typically been formulated considering the presence of CWBDs in Gram-positive infecting phage lysins (Loessner *et al.*, 2002 ▸), but may also be true in Gram-negative counterparts given that some of them contain motifs that are potentially toxic from without the cells, as demonstrated for Pae87.

Regarding the antimicrobial activity of peptide P87 itself, it was observed to cause an acute lytic effect on *P. aeruginosa* cells. The ability to form amphipathic helices in the presence of TFE, as demonstrated by the circular-dichroism spectra in Fig. 8 ▸, would point to the insertion of these helices into the biological membranes and subsequent leakage of the cell contents as the mechanism for P87-mediated killing. The peptide differs in this regard from the poor lytic outcome of Pae87 treatment. This difference may be due to the much smaller size of P87, which could enable it to properly insert into the membranes rather than interact superficially, as it is assumed that Pae87 does.

The maximum detectable killing was observed for 10 µ*M* P87 at cell concentrations of 10^7^ CFU ml^−1^ and below, while higher bacterial doses reduced the bactericidal effect, presumably due to a suboptimal number of antimicrobial molecules per cell. This is in agreement with a cooperative mechanism of action where a threshold number of bound peptides is required for bactericidal activity. In addition, the higher activity observed at more acidic pH values can be explained due to the higher positive charge that both the protein and peptide may have at acidic pH values, improving the interaction with the negatively charged bacterial surface. Given that an increased activity was observed at pH 6.0 and below, the protonation of histidine residues (p*K*_a_ ≃ 6.0) is the most plausible explanation. Although P87 was almost inactive at near-physiological pH (∼7.5), the fact that it was highly effective at acidic pH is relevant for the treatment of infection, since it has been suggested many times that the pH at the infection site is acidified by a combination of bacterial metabolic activity and immune-system responses (Radovic Moreno *et al.*, 2012 ▸; Simmen & Blaser, 1993 ▸). This acidification is especially relevant in certain conditions, such as cystic fibrosis, patients with which are already prone to infections in the respiratory tract, with *P. aeruginosa* being one of the main causative agents in the exacerbation of cystic fibrosis (Poschet *et al.*, 2002 ▸). The importance of charge is also manifested by the ionic strength experiments: P87 only displayed a relevant killing activity between 50 and 150 m*M* NaCl. It is possible that a slight salt concentration might be necessary for proper solubility of P87. Higher concentrations might, however, shield charged residues. This latter hypothesis is supported by the results in the presence of different concentrations of a non-ionic osmolyte (sorbitol), in which the killing activity of the peptide was not affected by an increasing concentration of the solute.

## 5. Conclusions

The three-dimensional structure of Pae87 has been elucidated by X-ray crystallography. This structure provided a basis to propose the presence of a substrate-binding subdomain within the catalytic domain of Pae87. This substrate-binding site is apparently conserved among other enzymes from the same family that lack an independent CWBD and thus may fulfil a compensatory evolutionary function. It was determined that Pae87 is a muramidase, and two acidic residues have been pointed out to be involved in this catalytic activity. However, the antimicrobial activity of Pae87, when exogenously added, was not associated with its catalytic activity, but rather with a nonenzymatic activity on the membranes that most probably resides on a cationic, amphiphilic C-terminal peptide named P87. Such a peptide was proven to be an AMP on its own, and was active against a range of bacteria coincidental to those susceptible to Pae87 surface activity. The activity of P87 was highly dependent on its intrinsic charge and on the peptide:bacteria stoichiometry. Altogether, these results provide further clarity on the structure of a family of Gram negative-active lysins, revealing evolutionary features with a close relationship to architectural traits of the bacterial hosts. On the other hand, insights have also been provided into the intrinsic antibacterial effect of Pae87 and a novel AMP (P87) has been discovered.

## 6. Related literature

The following reference is cited in the supporting information for this article: Artimo *et al.* (2012 ▸). 

## Supplementary Material

PDB reference: *Pseudomonas aeruginosa* bacteriophage JG004 endolysin Pae87, apo form, 7q4s

PDB reference: ligand-bound, 7q4t

Supplementary Tables S1 and S2. DOI: 10.1107/S2059798322000936/ni5018sup1.pdf

## Figures and Tables

**Table 1:** Crystallographic statistics of the Pae87 data sets Values in parentheses are for the highest resolution shell.

	Pae87	Pae87–peptidoglycan
Data collection		
Wavelength (Å)	0.97926	0.97933
Crystal-to-detector distance (mm)	488.7	212.5
Space group	*P*2_1_2_1_2_1_	*P*2_1_2_1_2_1_
*a*, *b*, *c* (Å)	59.25, 68.09, 93.10	43.58, 61.17, 69.21
Resolution range (Å)	59.25–2.50 (2.60–2.50)	69.21–1.27 (1.29–1.27)
Total No. of reflections	78046 (8657)	314568 (14575)
No. of unique reflections	13640 (1509)	49510 (2412)
Completeness (%)	99.7 (99.1)	99.9 (100.0)
Multiplicity	5.7 (5.7)	6.4 (6.0)
CC_1/2_ †	0.991 (0.860)	1.000 (0.903)
*R*_meas_ (all *I*+ and *I*−)‡	0.202 (1.087)	0.046 (0.828)
〈*I*/σ(*I*)〉	6.0 (2.1)	17.6 (2.0)
Wilson *B* factor (Å^2^)	31.8	13.9
Refinement		
Resolution range (Å)	55.02–2.50	45.85–1.27
Reflections used	12941	46988
Reflections used for *R*_free_	658	2454
*R* factor/*R*_free_ §	0.222/0.278	0.130/0.161
Model statistics		
No. of atoms		
Protein	2964	1506
Peptidoglycan	0	50
CHES	26	0
PEG	13	14
Ethylene glycol	0	52
Water	99	121
Average temperature factor (Å^2^)		
Protein	60.3	20.9
Peptidoglycan	—	50
CHES	89.0	—
PEG	73.4	42.2
Ethylene glycol	—	44.4
Water	41.4	35.1
Ramachandran statistics¶		
Favoured	96.5	97.8
Allowed	99.7	100.0
Disallowed	0.3	0.0
R.m.s.d., bond lengths†† (Å)	0.0016	0.0073
R.m.s.d., angles†† (°)	1.1	1.4
Clashscore/percentile‡‡	1.17/100th	2.76/97th
*MolProbity* score/percentile§§	1.06/100th	1.09/98th
PDB code	7q4s	7q4t

† CC_1/2_: correlation coefficient between intensity estimates from half data sets (Karplus & Diederichs, 2015 ▸).

‡ *R*_meas_ = 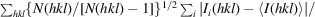 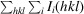 .

§ *R* = 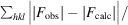 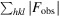 .

¶ Calculated by *MolProbity* (Williams *et al.*, 2018 ▸).

†† Calculated by *REFMAC*5 (Evans & Murshudov, 2013 ▸).

‡‡ The clashscore is the number of serious clashes per 1000 atoms, where the 100th percentile is the best among structures of comparable resolution and the 0th percentile is the worst.

§§ The *MolProbity* score combines the clashscore, rotamer and Ramachandran evaluations into a single score that is normalized to be on the same scale as the X-ray resolution (http://molprobity.biochem.duke.edu/; Williams *et al.*, 2018 ▸).

## References

[bb1] Abdelkader, K., Gerstmans, H., Saafan, A., Dishisha, T. & Briers, Y. (2019). *Viruses*, **11**, 96. 10.3390/v11020096 PMC6409994 30678377

[bb2] Alvarez, L., Hernandez, S. B., de Pedro, M. A. & Cava, F. (2016). *Methods Mol. Biol.* **1440**, 11–27. 10.1007/978-1-4939-3676-2_2 27311661

[bb60] Artimo, P., Jonnalagedda, M., Arnold, K., Baratin, D., Csardi, G., de Castro, E., Duvaud, S., Flegel, V., Fortier, A., Gasteiger, E., Grosdidier, A., Hernandez, C., Ioannidis, V., Kuznetsov, D., Liechti, R., Moretti, S., Mostaguir, K., Redaschi, N., Rossier, G., Xenarios, I. & Stockinger, H. (2012). *Nucleic Acids Res.* **40**, W597–W603. 10.1093/nar/gks400 PMC3394269 22661580

[bb3] Briers, Y. & Lavigne, R. (2015). *Future Microbiol.* **10**, 377–390. 10.2217/fmb.15.8 25812461

[bb4] Bublitz, M., Polle, L., Holland, C., Heinz, D. W., Nimtz, M. & Schubert, W. D. (2009). *Mol. Microbiol.* **71**, 1509–1522. 10.1111/j.1365-2958.2009.06619.x 19210622

[bb5] Dams, D. & Briers, Y. (2019). *Adv. Exp. Med. Biol.* **1148**, 233–253. 10.1007/978-981-13-7709-9_11 31482502

[bb6] Davies, G. & Henrissat, B. (1995). *Structure*, **3**, 853–859. 10.1016/S0969-2126(01)00220-9 8535779

[bb7] Duyvejonck, L., Gerstmans, H., Stock, M., Grimon, D., Lavigne, R. & Briers, Y. (2021). *Antibiotics*, **10**, 293. 10.3390/antibiotics10030293 PMC7998686 33799561

[bb8] Eckert, C., Lecerf, M., Dubost, L., Arthur, M. & Mesnage, S. (2006). *J. Bacteriol.* **188**, 8513–8519. 10.1128/JB.01145-06 PMC1698247 17041059

[bb9] Emsley, P., Lohkamp, B., Scott, W. G. & Cowtan, K. (2010). *Acta Cryst.* D**66**, 486–501. 10.1107/S0907444910007493 PMC2852313 20383002

[bb10] Evans, P. (2006). *Acta Cryst.* D**62**, 72–82. 10.1107/S0907444905036693 16369096

[bb11] Evans, P. R. & Murshudov, G. N. (2013). *Acta Cryst.* D**69**, 1204–1214. 10.1107/S0907444913000061 PMC3689523 23793146

[bb12] Gautier, R., Douguet, D., Antonny, B. & Drin, G. (2008). *Bioinformatics*, **24**, 2101–2102. 10.1093/bioinformatics/btn392 18662927

[bb13] Ghose, C. & Euler, C. W. (2020). *Antibiotics*, **9**, 74. 10.3390/antibiotics9020074 PMC7168136 32054067

[bb14] Groot, N. S. de, Castillo, V., Graña-Montes, R. & Ventura, S. (2012). *Methods Mol. Biol.* **819**, 199–220. 10.1007/978-1-61779-465-0_14 22183539

[bb15] Grütter, M. G. & Matthews, B. W. (1982). *J. Mol. Biol.* **154**, 525–535. 10.1016/s0022-2836(82)80011-9 7077670

[bb16] Guillén, D., Sánchez, S. & Rodríguez-Sanoja, R. (2010). *Appl. Microbiol. Biotechnol.* **85**, 1241–1249. 10.1007/s00253-009-2331-y 19908036

[bb17] Helander, I. M. & Mattila-Sandholm, T. (2000). *J. Appl. Microbiol.* **88**, 213–219. 10.1046/j.1365-2672.2000.00971.x 10735988

[bb18] Holm, L. (2020). *Protein Sci.* **29**, 128–140. 10.1002/pro.3749 PMC6933842 31606894

[bb19] Ibrahim, H. R., Higashiguchi, S., Koketsu, M., Juneja, L. R., Kim, M., Yamamoto, T., Sugimoto, Y. & Aoki, T. (1996). *J. Agric. Food Chem.* **44**, 3799–3806.

[bb20] Joosten, R. P., Long, F., Murshudov, G. N. & Perrakis, A. (2014). *IUCrJ*, **1**, 213–220. 10.1107/S2052252514009324 PMC4107921 25075342

[bb21] Juanhuix, J., Gil-Ortiz, F., Cuní, G., Colldelram, C., Nicolás, J., Lidón, J., Boter, E., Ruget, C., Ferrer, S. & Benach, J. (2014). *J. Synchrotron Rad.* **21**, 679–689. 10.1107/S160057751400825X PMC4073956 24971961

[bb22] Karplus, P. A. & Diederichs, K. (2015). *Curr. Opin. Struct. Biol.* **34**, 60–68. 10.1016/j.sbi.2015.07.003 PMC4684713 26209821

[bb23] Kezuka, Y., Ohishi, M., Itoh, Y., Watanabe, J., Mitsutomi, M., Watanabe, T. & Nonaka, T. (2006). *J. Mol. Biol.* **358**, 472–484. 10.1016/j.jmb.2006.02.013 16516924

[bb24] Krissinel, E. (2015). *Nucleic Acids Res.* **43**, W314–W319. 10.1093/nar/gkv314 PMC4489313 25908787

[bb25] Kuroki, R., Weaver, L. H. & Matthews, B. W. (1993). *Science*, **262**, 2030–2033. 10.1126/science.8266098 8266098

[bb26] Lacks, S. & Hotchkiss, R. D. (1960). *Biochim. Biophys. Acta*, **39**, 508–518. 10.1016/0006-3002(60)90205-5 14413322

[bb27] Loessner, M. J., Kramer, K., Ebel, F. & Scherer, S. (2002). *Mol. Microbiol.* **44**, 335–349. 10.1046/j.1365-2958.2002.02889.x 11972774

[bb28] Loh, B., Grant, C. & Hancock, R. E. (1984). *Antimicrob. Agents Chemother.* **26**, 546–551. 10.1128/aac.26.4.546 PMC179961 6440475

[bb29] Love, M. J., Coombes, D., Ismail, S., Billington, C. & Dobson, R. C. J. (2022). *Biochem. J.* **479**, 207–223. 10.1042/BCJ20210701 34935873

[bb30] Low, L. Y., Yang, C., Perego, M., Osterman, A. & Liddington, R. (2011). *J. Biol. Chem.* **286**, 34391–34403. 10.1074/jbc.M111.244160 PMC3190764 21816821

[bb31] Maciejewska, B., Źrubek, K., Espaillat, A., Wiśniewska, M., Rembacz, K. P., Cava, F., Dubin, G. & Drulis-Kawa, Z. (2017). *Sci. Rep.* **7**, 14501. 10.1038/s41598-017-14797-9 PMC5674055 29109551

[bb32] Madeira, F., Park, Y. M., Lee, J., Buso, N., Gur, T., Madhusoodanan, N., Basutkar, P., Tivey, A. R. N., Potter, S. C., Finn, R. D. & Lopez, R. (2019). *Nucleic Acids Res.* **47**, W636–W641. 10.1093/nar/gkz268 PMC6602479 30976793

[bb33] Mhlongo, N. N., Skelton, A. A., Kruger, G., Soliman, M. E. & Williams, I. H. (2014). *Proteins*, **82**, 1747–1755. 10.1002/prot.24528 24488819

[bb34] Murshudov, G. N., Skubák, P., Lebedev, A. A., Pannu, N. S., Steiner, R. A., Nicholls, R. A., Winn, M. D., Long, F. & Vagin, A. A. (2011). *Acta Cryst.* D**67**, 355–367. 10.1107/S0907444911001314 PMC3069751 21460454

[bb35] Ogata, M., Umemoto, N., Ohnuma, T., Numata, T., Suzuki, A., Usui, T. & Fukamizo, T. (2013). *J. Biol. Chem.* **288**, 6072–6082. 10.1074/jbc.M112.439281 PMC3585046 23303182

[bb36] O’Neill, J. (2016). *Tackling Drug-resistant Infections Globally: Final Report and Recommendations*. London: The Review on Antimicrobial Resistance. https://amr-review.org/sites/default/files/160525_Final%20paper_with%20cover.pdf.

[bb37] Pastagia, M., Schuch, R., Fischetti, V. A. & Huang, D. B. (2013). *J. Med. Microbiol.* **62**, 1506–1516. 10.1099/jmm.0.061028-0 23813275

[bb38] Poschet, J., Perkett, E. & Deretic, V. (2002). *Trends Mol. Med.* **8**, 512–519. 10.1016/s1471-4914(02)02414-0 12421684

[bb39] Radovic Moreno, A. F., Lu, T. K., Puscasu, V. A., Yoon, C. J., Langer, R. & Farokhzad, O. C. (2012). *ACS Nano*, **6**, 4279–4287. 10.1021/nn3008383 PMC3779925 22471841

[bb40] Rau, A., Hogg, T., Marquardt, R. & Hilgenfeld, R. (2001). *J. Biol. Chem.* **276**, 31994–31999. 10.1074/jbc.M102591200 11427528

[bb41] Ribes, S., Riegelmann, J., Redlich, S., Maestro, B., de Waal, B., Meijer, E. W., Sanz, J. M. & Nau, R. (2013). *Chemotherapy*, **59**, 138–142. 10.1159/000353439 24051739

[bb42] Rice, P., Longden, I. & Bleasby, A. (2000). *Trends Genet.* **16**, 276–277. 10.1016/s0168-9525(00)02024-2 10827456

[bb43] Rodríguez-Rubio, L., Gerstmans, H., Thorpe, S., Mesnage, S., Lavigne, R. & Briers, Y. (2016). *Appl. Environ. Microbiol.* **82**, 4975–4981. 10.1128/AEM.00446-16 PMC4968549 27287318

[bb44] Roig-Molina, E., Sánchez-Angulo, M., Seele, J., García-Asencio, F., Nau, R., Sanz, J. M. & Maestro, B. (2020). *ACS Infect. Dis.* **6**, 954–974. 10.1021/acsinfecdis.9b00344 32135064

[bb45] Sanz-Gaitero, M., Keary, R., Garcia-Doval, C., Coffey, A. & van Raaij, M. J. (2014). *Virol. J.* **11**, 133. 10.1186/1743-422X-11-133 PMC4126393 25064136

[bb46] Shannon, P., Markiel, A., Ozier, O., Baliga, N. S., Wang, J. T., Ramage, D., Amin, N., Schwikowski, B. & Ideker, T. (2003). *Genome Res.* **13**, 2498–2504. 10.1101/gr.1239303 PMC403769 14597658

[bb47] Simmen, H. P. & Blaser, J. (1993). *Am. J. Surg.* **166**, 24–27. 10.1016/s0002-9610(05)80576-8 8328625

[bb48] Thandar, M., Lood, R., Winer, B. Y., Deutsch, D. R., Euler, C. W. & Fischetti, V. A. (2016). *Antimicrob. Agents Chemother.* **60**, 2671–2679. 10.1128/AAC.02972-15 PMC4862495 26856847

[bb49] Vagin, A. & Teplyakov, A. (2010). *Acta Cryst.* D**66**, 22–25. 10.1107/S0907444909042589 20057045

[bb50] Vázquez, R., Blanco-Gañán, S., Ruiz, S. & García, P. (2021). *Front. Microbiol.* **12**, 660403. 10.3389/fmicb.2021.660403 PMC8185167 34113327

[bb51] Vázquez, R., García, E. & García, P. (2020). *Curated Phage Lysins Collection Including Identifiers, Amino Acid Sequences, Functional Domain Predictions, Architectures and Physicochemical Properties Calculations*. http://hdl.handle.net/10261/221469.

[bb52] Vázquez, R., García, E. & García, P. (2021). *J. Virol.* **95**, e0032121. 10.1128/JVI.00321-21 PMC8223927 33883227

[bb53] Waterhouse, A. M., Procter, J. B., Martin, D. M., Clamp, M. & Barton, G. J. (2009). *Bioinformatics*, **25**, 1189–1191. 10.1093/bioinformatics/btp033 PMC2672624 19151095

[bb54] Wilkins, M. R., Gasteiger, E., Bairoch, A., Sanchez, J. C., Williams, K. L., Appel, R. D. & Hochstrasser, D. F. (1999). *Methods Mol. Biol.* **112**, 531–552. 10.1385/1-59259-584-7:531 10027275

[bb55] Williams, C. J., Headd, J. J., Moriarty, N. W., Prisant, M. G., Videau, L. L., Deis, L. N., Verma, V., Keedy, D. A., Hintze, B. J., Chen, V. B., Jain, S., Lewis, S. M., Arendall, W. B., Snoeyink, J., Adams, P. D., Lovell, S. C., Richardson, J. S. & Richardson, J. S. (2018). *Protein Sci.* **27**, 293–315. 10.1002/pro.3330 PMC5734394 29067766

[bb56] Winn, M. D., Ballard, C. C., Cowtan, K. D., Dodson, E. J., Emsley, P., Evans, P. R., Keegan, R. M., Krissinel, E. B., Leslie, A. G. W., McCoy, A., McNicholas, S. J., Murshudov, G. N., Pannu, N. S., Potterton, E. A., Powell, H. R., Read, R. J., Vagin, A. & Wilson, K. S. (2011). *Acta Cryst.* D**67**, 235–242. 10.1107/S0907444910045749 PMC3069738 21460441

[bb57] Winter, G. (2010). *J. Appl. Cryst.* **43**, 186–190.

[bb58] Wohlkönig, A., Huet, J., Looze, Y. & Wintjens, R. (2010). *PLoS One*, **5**, e15388. 10.1371/journal.pone.0015388 PMC2976769 21085702

[bb59] Yang, H., Wang, D. B., Dong, Q., Zhang, Z., Cui, Z., Deng, J., Yu, J., Zhang, X. E. & Wei, H. (2012). *Antimicrob. Agents Chemother.* **56**, 5031–5039. 10.1128/AAC.00891-12 PMC3457386 22802245

[bb66] Zallot, R., Oberg, N. & Gerlt, J. A. (2019). *Biochemistry*, **58**, 4169–4182. 10.1021/acs.biochem.9b00735 PMC7057060 31553576

[bb61] Zimmermann, L., Stephens, A., Nam, S. Z., Rau, D., Kübler, J., Lozajic, M., Gabler, F., Söding, J., Lupas, A. N. & Alva, V. (2018). *J. Mol. Biol.* **430**, 2237–2243. 10.1016/j.jmb.2017.12.007 29258817

