# Supplementary material for: Monomodular Pseudomonas aeruginosa phage JG004 lysozyme (Pae87) contains a bacterial surface-active antimicrobial peptide-like region and a possible substrate-binding subdomain
Source: Acta Crystallogr D Struct Biol. 2022 Mar 4;78(Pt 4):435–54. doi: 10.1107/S2059798322000936 (PMC8972805; doi:10.1107/S2059798322000936)
Supplement: Supplementary file 1 [file d-78-00435-sup1.pdf]

STRUCTURAL BIOLOGY

Volume 78 (2022)

Supporting information for article:

Monomodular *Pseudomonas aeruginosa* phage JG004 lysozyme (Pae87) contains a bacterial surface-active antimicrobial peptide-like region and a possible substrate-binding subdomain

Roberto Vázquez, Mateo Seoane-Blanco, Virginia Rivero-Buceta, Susana Ruiz, Mark J. van Raaij and Pedro García

Table S1 . Plasmids and oligonucleotides used throughout this work.

Name	Description	Reference or source
	Plasmids	
pET-PA87	Derived from pET-28a(+) and pUCPA87, overexpresses gene *pae87* for production of protein Pae87, fused to a 6×His tag at N-terminal end. KAN[R]	(Vázquez *et al.*, 2021)
pET-PA87-E29	Derived from pET-PA87, overexpresses gene *pae87-e29* which encodes protein Pae87 with mutation E29A. KAN[R]	This work
pET-PA87-E46	Derived from pET-PA87, overexpresses gene *pae87-e46* which encodes protein Pae87 with mutation E46A. KAN[R]	This work
pET-PA87-E2946	Derived from pET-PA87-E29, overexpresses gene *pae87-e2946* which encodes protein Pae87 with mutations E29A and E46A. KAN[R]	This work
	Oligonucleotides (5' → 3')	
pae87_f	CTAAGGTACCCATATGGCTCTGACCGAGCAAGACTTCC	5' of *pae87* (forward)
pae87_3'	TACAAAGCTTATTTGAAGGATTGATAGGCTTCTGCCAG	3' of *pae87* (reverse)
e29a_f	CGTCACCAAAGTAGCGAGTCGTGGG	Triplet coding for E29 of *pae87*, for mutation (forward)
e29a_r	CCCACGACTCGCTACTTTGGTGACG	Triplet coding for E29 of *pae87*, for mutation (reverse)
e46a_f	TTCTGTTCGCACGCCACTGG	Triplet coding for E46 of *pae87*, for mutation (forward)
e46a_r	TGGCGTGCGAACAGAATTTTCGG	Triplet coding for E46 of *pae87*, for mutation (reverse)

Restriction enzyme recognition sites are underlined and mutated bases with respect to the wild-type sequence are highlighted in grey.

Table S2 Protein parameters as predicted by ProtParam (Artimo *et al.*, 2012).

Protein	Molecular mass (kDa)	Number of aa	p*I*	Molar extinction coefficient (M^{-1} cm^{-1})
Pae87	23.05	206	9.11	32555
E29A	22.99	206	9.24	32555
E46A	22.99	206	9.24	32555
E29A E46A	22.93	206	9.35	32555

Artimo, P., Jonnalagedda, M., Arnold, K., Baratin, D., Csardi, G., de Castro, E., Duvaud, S., Flegel, V., Fortier, A., Gasteiger, E., Grosdidier, A., Hernandez, C., Ioannidis, V., Kuznetsov, D., Liechti, R., Moretti, S., Mostaguir, K., Redaschi, N., Rossier, G., Xenarios, I. & Stockinger, H. (2012). *Nucleic Acids Res* **40**, W597-603.
Vázquez, R., Blanco-Gañán, S., Ruiz, S. & García, P. (2021). *Front Microbiol* **12**, 660403.
